# Norepinephrine promotes triglyceride storage in macrophages via beta2‐adrenergic receptor activation

**DOI:** 10.1096/fj.202001101R

**Published:** 2021-01-23

**Authors:** Kasparas Petkevicius, Guillaume Bidault, Sam Virtue, Benjamin Jenkins, Xanthe A. M. H. van Dierendonck, Aurelien Dugourd, Julio Saez‐Rodriguez, Rinke Stienstra, Albert Koulman, Antonio Vidal‐Puig

**Affiliations:** ^1^ Institute of Metabolic Science MDU MRC University of Cambridge Metabolic Research Laboratories Cambridge United Kingdom; ^2^ Nutrition, Metabolism and Genomics Group Division of Human Nutrition and Health Wageningen University Wageningen the Netherlands; ^3^ Department of Internal Medicine Radboud University Medical Center Nijmegen the Netherlands; ^4^ Joint Research Centre for Computational Biomedicine Faculty of Medicine RWTH Aachen University Aachen Germany; ^5^ Institute for Computational Biomedicine Faculty of Medicine & Heidelberg University Hospital Heidelberg University Heidelberg Germany; ^6^ Wellcome Trust Sanger Institute Hinxton United Kingdom; ^7^ Present address: Metabolism Bioscience, Cardiovascular, Renal and Metabolism IMED Biotech Unit AstraZeneca BioPharmaceuticals R&D Gothenburg Sweden

**Keywords:** adrb2, dgat1, hilpda, immunometabolism, lipid

## Abstract

Tissue‐resident macrophages are required for homeostasis, but also contribute to tissue dysfunction in pathophysiological states. The sympathetic neurotransmitter norepinephrine (NE) induces an anti‐inflammatory and tissue‐reparative phenotype in macrophages. As NE has a well‐established role in promoting triglyceride lipolysis in adipocytes, and macrophages accumulate triglyceride droplets in various physiological and disease states, we investigated the effect of NE on primary mouse bone marrow‐derived macrophage triglyceride metabolism. Surprisingly, our data show that in contrast to the canonical role of NE in stimulating lipolysis, NE acting via beta2‐adrenergic receptors (B2ARs) in macrophages promotes extracellular fatty acid uptake and their storage as triglycerides and reduces free fatty acid release from triglyceride‐laden macrophages. We demonstrate that these responses are mediated by a B2AR activation‐dependent increase in *Hilpda* and *Dgat1* gene expression and activity. We further show that B2AR activation favors the storage of extracellular polyunsaturated fatty acids. Finally, we present evidence that macrophages isolated from hearts after myocardial injury, for which survival critically depends on leukocyte B2ARs, have a transcriptional signature indicative of a transient triglyceride accumulation. Overall, we describe a novel and unexpected role of NE in promoting triglyceride storage in macrophages that could have potential implications in multiple diseases.

**Abbreviations**
ADRP: adipose differentiation‐related protein
ATGL: adipose triglyceride lipase
B2AR: β2‐adrenergic receptor
BMDM: bone marrow‐derived macrophage
BSA: bovine serum albumin
cAMP: cyclic adenosine monophosphate
CRE: cAMP‐responsive element
CREB: cAMP‐responsive element‐binding protein
DAG: diacylglycerol
DGAT: diacylglycerol acyltransferase
FFA: free fatty acid
HILPDA: hypoxia‐inducible lipid droplet associated
LPS: lipopolysaccharide
NE: norepinephrine
PC: phosphatidylcholine
PE: phosphatidylethanolamine
PS: phosphatidylserine
qPCR: quantitative polymerase chain reaction
ROS: reactive oxygen species
TG: triglyceride

## 1 INTRODUCTION

Macrophages are innate immune cells that reside in all mammalian tissues. In a healthy tissue, macrophages are involved in processes related to tissue homeostasis, such as phagocytosis of apoptotic cells and the turnover of extracellular matrix.^1^ Moreover, macrophages are essential for the restoration of tissue homeostasis following an injury or infection—macrophages are rapidly recruited to the site of injury, where their pro‐inflammatory activation allows the phagocytosis of dying cells and invading pathogens. Once the initial insult has been resolved, macrophages subsequently acquire an anti‐inflammatory phenotype that is necessary for wound healing and tissue repair.^1^ Such macrophage activity within tissues is tightly regulated by a multitude of damage‐ and pathogen‐associated molecules, as well as signaling factors from other tissue‐resident cells.^2^ Abnormal macrophage phenotypes are observed in nearly all human pathophysiological states, and macrophages contribute to the progression of many diseases, such as type‐2 diabetes, atherosclerosis, and cancer.^3^ Therefore, there is a major interest in understanding how macrophage behavior is regulated in various disease states, and whether these regulatory pathways can be targeted therapeutically.

One of the regulators of macrophage activity within tissues is the sympathetic nervous system. Sympathetic nerve endings within tissues secrete the neurotransmitter norepinephrine (NE) upon firing, which then binds to the adrenergic receptors in tissue‐resident cells. NE has been shown to predominantly signal to macrophages via beta2‐adrenergic receptor (B2AR).^4^ In adipose tissues, sympathetic nerve activity maintains resident macrophages in an anti‐inflammatory state in a B2AR‐signalling dependent manner.^5^, ^6^ Similarly, B2AR activation by sympathetic neurons in the small intestine during enteric infection is critical to drive a tissue‐protective macrophage phenotype.^7^ The loss of gut sympathetic innervation elicits an innate immune system‐driven colitis,^8^ and the deletion of macrophage B2ARs exacerbates neuronal damage following enteric infection.^9^ Similarly, pharmacological blockade of B2AR enhances lipopolysaccharide (LPS)‐induced lung injury in mice.^10^ Furthermore, B2AR signaling in macrophages is necessary for heart repair and survival after myocardial infarction.^11^, ^12^ However, while the NE‐B2AR signaling axis has been described to play anti‐inflammatory and tissue‐protective roles in macrophages in multiple settings, other potential roles for NE signaling in macrophages are yet to be fully investigated.

One of the best‐described roles of the sympathetic nervous system in other physiological contexts is the stimulation of lipolysis in adipocytes during states of negative energy balance, such as fasting.^13^ NE binding to the adipocyte beta‐adrenergic receptors leads to the intracellular activation of protein kinase A (PKA), which phosphorylates lipid droplet protein perilipin‐1.^13^ Phosphorylated perilipin‐1 allows adipose triglyceride lipase (ATGL) to access triglycerides stored within lipid droplets and hydrolyze them to release free fatty acids (FFAs).^13^ Therefore, NE has a major role in regulating cellular triglyceride metabolism.

Lipid droplet‐containing macrophages have been observed in various physiological and pathological states. In white adipose tissues, resident macrophages accumulate triglycerides as lipid droplets after fasting‐ or pharmacologically induced lipolysis.^14^, ^15^ Obesity also leads to an increase in the population of lipid‐laden adipose tissue macrophages, which coincides with the development of adipose tissue inflammation and insulin resistance.^16^, ^17^ Similarly, cholesterol‐rich lipid droplet containing macrophages are found in atherosclerotic plaques.^18^ Furthermore, the accumulation of triglyceride‐rich lipid droplets in brain‐resident macrophages (microglia) during aging has been shown to contribute to neurodegeneration,^19^ and increased triglyceride storage in tumor‐associated macrophages has been linked to cancer progression.^20^, ^21^ Finally, macrophage pro‐inflammatory activation by bacterial components and/or interferon‐gamma leads to increased fatty acid partitioning to triglycerides due to the near‐complete inhibition of mitochondrial fatty acid oxidation that happens in response to inflammatory activation.^22^, ^23^, ^24^, ^25^, ^26^, ^27^

The role of neutral lipid storage in lipid droplets in macrophages is not clear. Some reports have shown that increased neutral lipid storage by macrophages reduces the intracellular levels of reactive pro‐inflammatory lipid intermediates, and thus plays an anti‐inflammatory role.^20^, ^21^, ^28^, ^29^ In support of this, macrophage‐specific overexpression of the gene‐encoding Diacylglycerol O‐Acyltransferase 1 (DGAT1), which catalyzes the final step in triglyceride biosynthesis, alleviates adipose tissue inflammation and improves systemic insulin sensitivity in mice fed high‐fat diet.^29^ Others have demonstrated that lipid droplets can act as reservoirs for precursor molecules required for pro‐inflammatory mediator synthesis, and therefore increased macrophage triglyceride storage can heighten inflammatory response.^30^, ^31^, ^32^ Support for the lipid‐mediator pool hypothesis comes from data obtained from macrophages lacking Hypoxia Inducible Lipid Droplet Associated (HILPDA) protein, a potent endogenous inhibitor of ATGL.^33^ *Hilpda*‐deficient macrophages cannot store triglycerides, leading to impaired prostaglandin E2 production.^30^ Reduced prostaglandin secretion in turn translates into reduced atherosclerotic plaque size and inflammation in macrophage‐specific *Hilpda* knockout mice compared to controls on an ApoE^‐/‐^ genetic background.^30^ Finally, it has also been shown that triglyceride storage in lipid droplets in activated macrophages can be disassociated from their inflammatory phenotypes^15^, ^34^—adipose tissue macrophages isolated from high‐fat diet‐fed macrophage‐specific *Hilpda* knockout mice have no lipid droplets, but the development of adipose tissue inflammation and insulin resistance in these mice are comparable to controls.^34^ Notably, while lipid‐laden macrophages have been identified in multiple physiological and disease states, and such lipid accumulation has been either positively or negatively linked to disease progression, the endocrine and paracrine signaling factors that specifically regulate lipid storage in macrophages are yet to be identified.

Given the central role of NE in stimulating triglyceride lipolysis in adipocytes, here we investigated the role of adrenergic signaling on lipid metabolism in mouse primary bone marrow‐derived macrophages (BMDMs). We found that B2AR activation in BMDMs rapidly upregulated the transcription of genes involved in triglyceride biosynthesis, namely, *Dgat1* and *Hilpda*. We then showed that B2AR stimulation in macrophages increased the storage of extracellular fatty acids as triglycerides, and that this effect was mediated by increased DGAT1 activity and by partial ATGL inhibition, likely due to increased HILPDA expression. Finally, we used data from mouse models of heart injury, which is characterized by a rapid surge in sympathetic nerve activity^35^ and requires macrophage B2ARs for the tissue repair and survival.^11^ We indicated that the transcriptional profile of increased triglyceride storage is also observed in macrophages isolated from injured hearts, suggesting that NE‐B2AR signaling might contribute to the triglyceride accumulation in macrophages in vivo.

## 2 MATERIALS AND METHODS

### 2.1 Reagents

All fatty acids and A922500 (10012708) were from Cayman. *E coli* LPS (L4391), (±)‐Norepinephrine (+)‐ bitartrate salt (A0937), fenoterol hydrobromide (F1016), bafilomycin A1 (B1793), and atglistatin (SML1075) were from Sigma. Interleukin‐4 (214‐14) was from Peprotech. ICI‐118,511 (0821) was from Tocris.

### 2.2 Mice

This research has been regulated under the Animals (Scientific Procedures) Act 1986 Amendment Regulations 2012 following ethical review by the University of Cambridge Animal Welfare and Ethical Review Body (AWERB). Mice used for generation of BMDMs were male on a C57Bl/6J genetic background (bred in‐house or bought from Charles River, UK), used between 8 and 20 weeks of age. Macrophage‐specific *Adrb2* knockout mouse was generated by crossing a mouse model containing *loxP* sequences surrounding *Adrb2* alleles (*Adrb2* ^fl/fl^) to the Lyz2‐Cre mouse. *Adrb2* ^fl/fl^ mouse was generated by Prof. Florent Elefteriou and Prof. Gerard Karsenty as described^36^ and was kindly gifted to us by Prof. Gerard Karsenty on a pure C57Bl/6J genetic background. Hilpda^fl/fl^ and Hilpda^fl/fl^ Lyz2‐Cre mice were generated and bred as previously described.^34^

### 2.3 Culture of bone marrow–derived macrophages

Freshly isolated bone marrow cells were seeded in RPMI‐1640 with 20% L929 conditioned medium (generated as described^37^), 10% heat‐inactivated FBS (Gibco, Thermo Fisher Scientific), and 100 U/mL penicillin‐streptomycin (Thermo Fisher Scientific) in 10 cm non‐culture treated plates (Falcon) at a density of 5 × 10^6^ cells per plate per 10 mL of medium and cultured for 7 days at 37℃ in 5% CO_2_. Medium was replaced to a fresh one on day 5. On day 7, BMDMs were detached using ice‐cold PBS containing 1 mM EDTA, seeded in macrophage differentiation medium in cell culture plates (Falcon) at the density of 1.35 × 10^6^ cells/cm^2^, and used for experiments the following day. The cellular identity of BMDMs was routinely verified by measuring the expression of macrophage surface markers by flow cytometry as previously described^37^ (Figure S1).

### 2.4 Cell stimulations

All BMDM stimulations were performed in complete macrophage differentiation medium. Fatty acid treatments were done using FFAs conjugated to BSA (A8806, Sigma). 5% BSA solution in macrophage differentiation medium was conjugated with 2.5 mM fatty acid as described.^37^ In an experiment involving FFA mixtures, FFAs were conjugated to 5% BSA at 5 mM concentration (higher concentration was chosen to increase the abundance of intracellular triglycerides, in order to maximize the signal‐to‐noise ratio for LC‐MS detection of triglyceride species or for Oil Red O staining). For radiolabeling experiments, 0.006 MBq/mL [1‐^14^C]‐oleic acid (NEC317050UC, Perkin Elmer) was used together with non‐labeled oleic acid. For stable labeling experiment, pure [1‐^13^C]‐oleic acid (CLM‐149, Cambridge Isotope Laboratories) was used at an indicated concentration. In co‐stimulation experiments, inhibitors were added 30‐45 minutes prior to B2AR activation or FFA treatment, and B2AR agonists were added 5 minutes prior to FFA treatment.

### 2.5 Extraction of RNA and subsequent qPCR

RNA was extracted using Rneasy Plus Mini kit (74106, Qiagen) following manufacturers' instructions. Thirty μL of RNAse‐free water was used for elution. Complementary DNA (cDNA) was generated from 250 ng RNA using Promega M‐MLV reverse transcriptase kit in a 20‐μL reaction according to manufacturer's instructions. cDNA was diluted 75‐fold in RNAse‐free water and stored at −20°C. qRT‐PCR was performed using TaqMan or SYBR Green reagents according to manufacturer's instructions (Applied Biosystems). A standard curve generated from a pool of all cDNA samples was used for quantification. The expression of genes of interest was normalized using BestKeeper method to the geometric average of 3‐4 housekeeping genes (*18s, 36b4, Actb,* and *Tbp*), and data were expressed as arbitrary units. Primer sequences (5′‐3′) used in this study are listed in Table 1.

**TABLE 1:** qPCR primers used in this study

Gene	Forward primer (5′‐3′)	Reverse primer (5′‐3′)	Probe (5′‐3′)
*18s*	CGGCTACCACATCCAAGGAA	GCTGGAATTACCGCGGCT	GAGGGCAAGTCTGGTGCCAG
*36b4*	AGATGCAGCAGATCCGCAT	GTTCTTGCCCATCAGCACC	
*Actb*	GCTCTGGCTCCTAGCACCAT	GCCACCGATCCACACAGAGT	ATCAAGATCATTGCTCCTCCTGAGCGC
*Adrb2*	TGGTGGTGATGGTCTTTGTC	GTCTTGAGGGCTTTGTGCTC	
*Ptgs2*	CCCTGAAGCCGTACACATCA	GTCACTGTAGAGGGCTTTCAATTCT	TTGAAGAACTTACAGGAGAGAAGGAAATGGCTG
*Dgat1*	AGGTTCTCTAAAAATAACCTTGCATT	TCGTGGTATCCTGAATTGGTG	
*Dusp1*	GTGCCTGACAGTGCAGAATC	CACTGCCCAGGTACAGGAAG	
*Hilpda*	GCACGACCTGGTGTGACTGT	CCAGCACATAGAGGTTCAGCAT	
*Klf4*	CGGGAAGGGAGAAGACACT	GAGTTCCTCACGCCAACG	
*Nr4a1*	CTGTCCGCTCTGGTCCTC	AATGCGATTCTGCAGCTCTT	
*Tbp*	CAAACCCAGAATTGTTCTCCTT	ATGTGGTCTTCCTGAATCCCT	

### 2.6 RNA sequencing

One µg of total RNA was quality checked (RIN > 8.5) using an Agilent Bioanalyser 2100 system and then used to construct barcoded sequencing libraries with Illumina's TruSeq Stranded mRNA Library Prep Kit following manufacturer's instruction. All the libraries where then multiplexed and sequenced on one lane of Illumina HiSeq 4000 at SE50 at CRUK Cambridge Institute Genomics Core Facility. Sequence reads were mapped onto the GRCm38 genome using TopHat v2.0.11 and genes were counted using HTseq‐count (V0.8.0). Statistical analysis of the counts was performed in R (build 3.4.1). Counts were log_2_ normalized using Variance Normalization Stabilization (vsn). Differential expression analysis was performed using limma library and pathway analysis was performed using PIANO packages (used with all base enrichment methods except Wilcoxon, with 10 000 permutations if possible). Raw NGS data and a matrix containing raw gene counts have been deposited in GEO, accession number GSE160640.

### 2.7 Western blotting

BMDM protein lysates were subjected to SDS‐PAGE 10% polyacrylamide electrophoresis (NuPAGE, Thermofisher) and transferred onto a nitrocellulose membrane (iBlot, Thermofisher) according to manufacturer's protocol. Membranes were blocked for 1 hour in 5% milk (Marvel) and probed with the following antibodies overnight at 4°C: anti‐CREB (48H2, Cell signaling), anti‐P‐Ser133 CREB (87G3, Cell signaling), and anti‐beta‐actin (ab8227, Abcam), then detected using a peroxidase‐coupled secondary anti‐rabbit antibody (7074, Cell signaling) and enhanced chemiluminescence (WBLUF0500, Millipore).

### 2.8 DGAT activity assay

Microsomes were isolated from 10^7^ BMDMs as described.^38^ Briefly, cells were homogenized in 5 mL of homogenizing buffer (10 mM Tis‐HCl, 0.25 M sucrose, 1 mM EDTA, pH 7.0) and centrifuged as follows: 10 minutes at 700 g, 10 minutes at 8000 g, and 10 minutes at 17 000 g, all at 4°C. The supernatant was then transferred to a fresh tube and centrifuged at 1 05 000 g, 4°C for 45 minutes. The pellet containing microsomes was resuspended in 50 μL of 0.1 M Tris‐HCl (pH 7.4). Microsomal DGAT activity was determined by measuring the esterification of palmitoyl‐CoA and oleoyl‐CoA to sn‐1,2‐dioctanoylglycerol in the same reaction by GC‐MS as described^39^ and normalized to total microsomal protein concentration.

### 2.9 Oil Red O staining

Oil Red O staining was performed following the standard protocol as described.^34^ In order to quantify the Oil Red O staining intensity, images were first adjusted for white balance, then red channel was isolated, and the percentage of dark pixels in the image was quantified after adjusting the threshold against the background.

### 2.10 Fatty acid oxidation assay

FAO assay was adapted from a published protocol.^40^ BMDMs were treated as described in the legend before washing them with PBS and incubating in the presence of FAO medium (DMEM containing 12.5 mM HEPES, 1 mM L‐carnitine, 0.3% BSA, and 100 μM oleate with ^14^C‐tracer). Zero h NE and etomoxir treatments were performed immediately after the medium change. Plates were then immediately sealed with parafilm and placed at 37°C for 3 hours. Once the FAO reaction finished, 400 μL of the reaction medium was transferred into the CO_2_ trapping tubes containing 200 μL of concentrated HCl and NaOH‐containing paper discs in the lids, then incubated for 1 hour at room temperature. ^14^CO_2_ was detected by measuring the radioactivity in the paper disks by liquid scintillation counting.

### 2.11 Seahorse mitochondrial stress assay

BMDMs were plated in XF‐96 cell culture plates (Agilent) at a density of 50 000 cells per well, and the cells were stimulated with 1 μM NE at 1 or 4 hours prior to the assay. Cells were washed and the medium change for the XF assay medium (unbuffered DMEM pH 7.4 supplemented with 2 mM L‐glutamine and 11 mM glucose), then incubated for 1 hour in a non‐CO2 incubator, following manufacturer instructions. Real‐time measurement of oxygen‐consumption rate (OCR) was performed using an XF‐96 Extracellular Flux Analyzer (Seahorse Bioscience). To assess mitochondrial respiration, four consecutive measurements were obtained under basal conditions and after the sequential addition of 1 μM oligomycin, to inhibit mitochondrial ATP synthase; 1.5 μM FCCP (fluoro‐carbonyl cyanide phenylhydrazone), a protonophore that uncouples ATP synthesis from oxygen consumption by the electron‐transport chain; 40 μM Etomoxir, a CPT1a inhibitor to inhibit fatty acid oxidation, and 100 nM rotenone plus 1 μM antimycin A, which inhibit the electron transport chain. All drugs were purchased from Sigma.

### 2.12 Lipid extraction

Total lipids from cells were extracted using a modified Folch extraction method as described,^37^ dried under nitrogen stream, and stored at −20°C for subsequent processing. Neutral lipid fraction was isolated in chloroform using solid‐phase extraction columns (Bond Elut, 12113014, Agilent).

### 2.13 Thin‐layer chromatography

Dried lipids from 10^6^ BMDMs were solubilized in 50 µL of chloroform. Twenty µL of lipids were then spotted at the bottom of TLC silica plates (Z292974, Sigma). TLC plates were placed into hermetic glass chambers containing one of the following solutions: 65:25:4 chloroform:methanol:ammonium hydroxide v/v for phospholipid separation, or 80:20:1 hexane:diethyl ether:acetic acid v/v for neutral lipid separation. Plates were developed, dried under laminar flow, and incubated with radiographic films (47410, Fujifilm) overnight at room temperature. Radiographic films were developed using automated film developer and scanned. ImageJ software (NIH) was used to calculate the density of the bands on scanned radiograms.

### 2.14 [1‐^13^C]‐oleic acid detection by GC‐MS

Dried neutral lipids from 10^7^ BMDMs were hydrolyzed and converted into fatty acid methyl esters, which were then detected by GC (Agilent 7890B) coupled to MS (Agilent 5977A) using a TR‐FAME column (260M142P, Thermofisher) as described.^37^ 1‐^13^C oleate was quantified from an M + 1 ion of ^12^C‐oleate‐methyl ester (285 m/z).

### 2.15 LC‐MS lipid analysis

To the previously dried lipid samples, 60 µL of the lipid internal standard was added (methanol containing CE(18:0)d6, Ceramide(16:0)d31, FA(15:0)d29, LPC(14:0)d42, PA(34:1)d31, PC(34:1)d31, PE(34:1)d31, PG(34:1)d31, PI(34:1)d31, PS(16:0)d62, SM(16:0)d31, TG(45:0)d29, and TG(54:0)d35, all at 10 µg/mL). The samples were then thoroughly vortexed, then dried under a gentle stream of nitrogen. The samples were then reconstituted by adding 740 µL of 4:1 mix of isopropanol and acetonitrile, respectively, and vortexed ensuring there was no undissolved material. The samples were then analyzed by LC‐MS as described.^37^ The semi‐quantitation of the lipids was calculated by the integration of the analyte MS signal relative to the lipid class internal standard concentration.

### 2.16 Statistical analysis

All data from experiments is represented as a mean, with error bars showing standard error of the mean and the number of replicates stated in the legend. Some data are represented as a fold change, and it is stated in legend to what value the data represented was normalized to generate the fold change. Statistical tests used are also stated in legend. All statistical tests were performed and graphs were generated using GraphPad Prism 6 software. In order to assess similarities between RNA sequencing datasets, we used GSEA to see if the DEG by fenoterol in BMDMs were significantly enriched in the top 500 DEG of a heart injury macrophage dataset, and vice versa. Graphs and figures were edited for presentation using Adobe Illustrator CC 2015 software.

## 3 RESULTS

### 3.1 B2AR stimulation rapidly upregulates CREB‐dependent gene transcription in BMDMs

**FIGURE 1:**
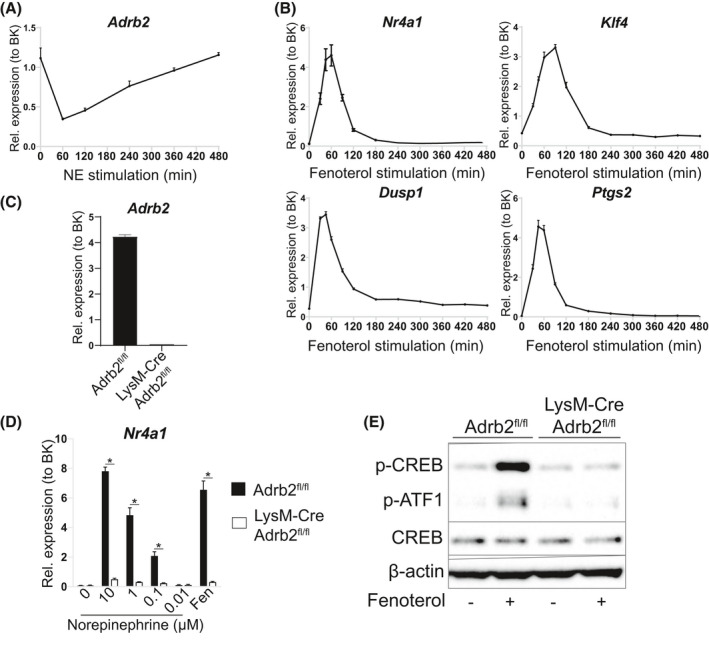
NE stimulates CREB‐dependent gene expression via B2ARs in BMDMs. A, *Adrb2* mRNA levels in BMDMs treated with 1 μM NE for indicated periods of time. B, *Nr4a1, Klf4, Dusp1,* and *Ptgs2* mRNA levels in BMDMs treated with 1 μM fenoterol for indicated periods of time. C, *Adrb2* mRNA levels in BMDMs from *Adrb2* ^fl/fl^ and Lyz2‐Cre *Adrb2* ^fl/fl^ mice. D, *Nr4a1* mRNA levels in *Adrb2* ^fl/fl^ and Lyz2‐Cre *Adrb2* ^fl/fl^ BMDMs treated with indicated doses of NE or 1 μM fenoterol for 1 h. E, Representative Western blot of indicated proteins in *Adrb2* ^fl/fl^ and Lyz2‐Cre *Adrb2* ^fl/fl^ BMDMs treated with 1 μM fenoterol for 20 min. All graphs show means ± SEM. N = 4 mice per treatment or genotype in all experiments. In D, *indicates *P* < .05 compared between genotypes using two‐way ANOVA with Bonferroni's multiple comparisons test. In A‐C, significant differences were not assessed

Prior to investigating the effects of NE on BMDM lipid metabolism, we sought to understand the specificity and kinetics of NE signaling in macrophages. Given the extensive literature on the importance of B2AR in macrophage biology, we focused on B2AR as the main receptor of NE in BMDMs. Indeed, the *Adrb2* gene that encodes B2AR was the highest expressed adrenergic receptor isoform in mouse BMDMs (data not shown). *Adrb2* expression was rapidly downregulated following stimulation with NE (Figure 1A), indicating a negative feedback loop that could act to limit further signaling by reducing receptor expression. We then focused on B2AR‐induced downstream PKA activation. One of the best‐described PKA phosphorylation targets is a transcription factor cAMP response element‐binding protein (CREB), which activates the transcription of genes with CRE sequences in their promoter.^41^ In order to monitor CREB activity, we measured the expression levels of four genes, namely, *Nr4a1*, *Klf4*, *Dusp1,* and *Ptgs2*, that have established roles in macrophage biology and are *bona fide* CREB targets.^41^ Stimulating BMDMs with fenoterol, a B2AR‐specific agonist, rapidly induced the transcription of all four CREB‐dependent genes, with peak expression levels observed 1 hour post stimulation (Figure 1B).

In order to understand whether NE stimulates gene expression specifically via B2ARs, we obtained BMDMs from macrophage‐specific *Adrb2* knockout mice. These mice had previously been described to have unaltered macrophage development, thus represented a valid model for our experiments.^9^ We observed an almost complete loss of *Adrb2* expression in *Adrb2* knockout BMDMs (Figure 1C). Importantly, NE induced the expression of *Nr4a1* in WT BMDMs in a dose‐dependent manner, and this increase was almost completely abolished in *Adrb2* knockout cells (Figure 1D). Finally, we found that fenoterol induced PKA activation in WT macrophages, as evidenced by increased CREB phosphorylation on PKA target phosphosite Ser133, and this activation was abolished in *Adrb2* knockout BMDMs (Figure 1E). Overall, we found that NE rapidly activated CREB‐dependent gene transcription in BMDMs in a B2AR‐specific manner.

### 3.2 B2AR stimulation upregulates the transcription of genes involved in triglyceride storage in BMDMs

**FIGURE 2:**
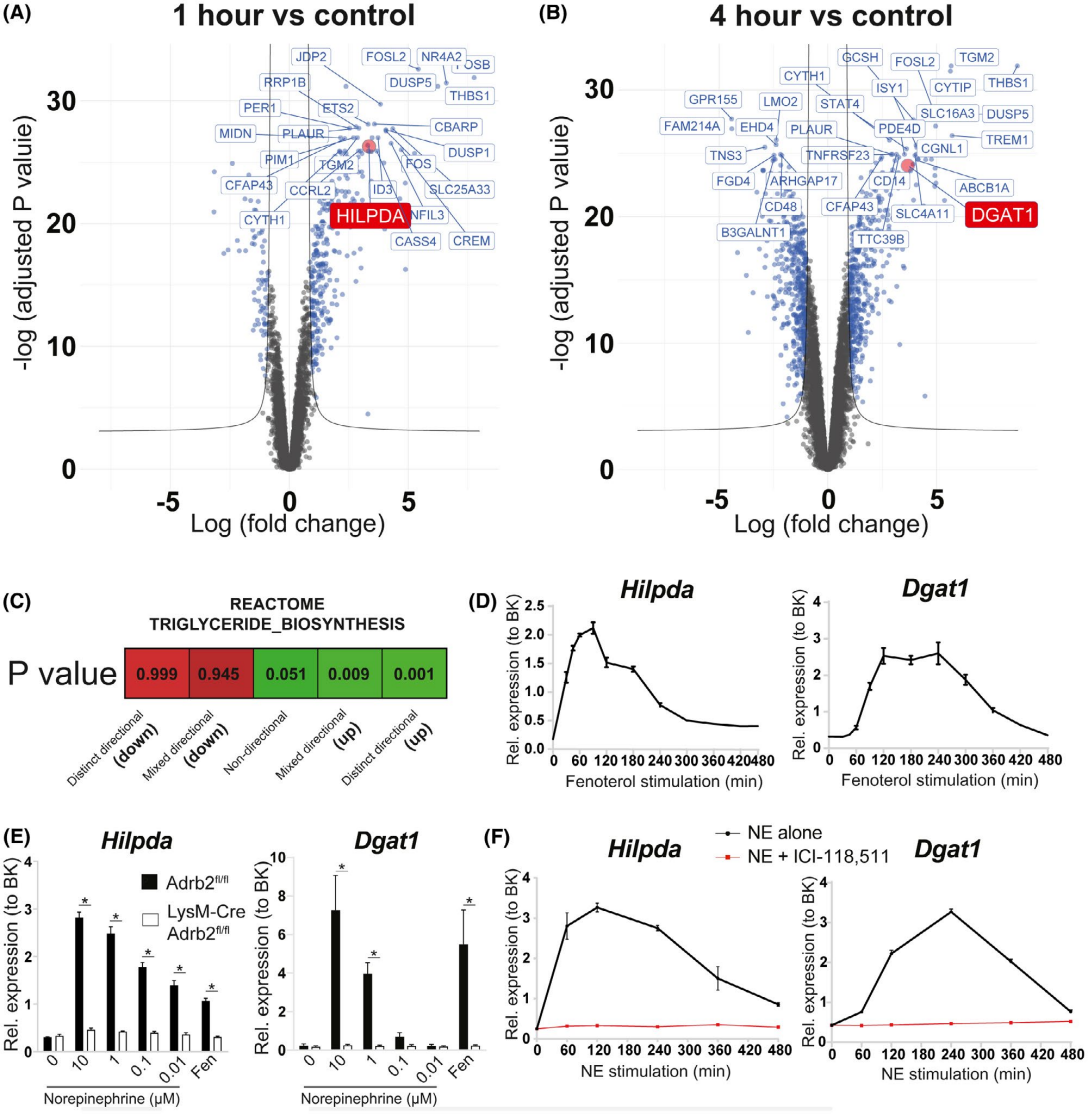
B2AR activation in BMDMs promotes the transcription of genes involved in triglyceride biosynthesis. Volcano plots indicating the differentially expressed genes as blue dots between BMDMs treated with 1 μM fenoterol for A) 1 h or B) 4 h and untreated controls. C, ‘Reactome_Triglyceride_Biosynthesis' pathway activation status based on the changes in global gene expression between BMDMs treated with 1 μM fenoterol for 1 h and untreated controls. D, *Hilpda and Dgat1* mRNA levels in BMDMs treated with 1 μM fenoterol for indicated periods of time. E, *Hilpda and Dgat1* mRNA levels in *Adrb2* ^fl/fl^ and Lyz2‐Cre *Adrb2* ^fl/fl^ BMDMs treated with indicated doses of NE or 1 μM fenoterol for 1 h (*Hilpda*) or 4 h (*Dgat1*). F, *Hilpda* and *Dgat1* mRNA levels in BMDMs treated with 1 μM NE in the presence or absence of 1 μM B2AR antagonist ICI‐118,511 for indicated periods of time. All graphs show means ± SEM. N = 4 mice per treatment or genotype in all experiments. In E, *indicates *P* < .05 compared between genotypes using two‐way ANOVA with Bonferroni's multiple comparisons test. In D, F, significant differences were not assessed

Having established the specificity and kinetics of NE signaling in BMDMs, we decided to perform global transcriptomic analysis in order to identify transcriptional changes in response to fenoterol stimulation. We chose two timepoints following B2AR activation—1 hour, representing a peak expression levels of the upregulated CREB‐dependent genes (Figure 1B), and 4 hours, representing any potential genes with delayed transcriptional activation. RNA sequencing analysis of the two timepoints of activation showed two distinct profiles of transcriptional regulation—at 1 hour, B2AR stimulation predominantly resulted in the upregulation of gene transcription (Figure 2A), while at 4 hours there were comparable numbers of increased and decreased differentially expressed genes (DEGs) (Figure 2B).

Interestingly, further bioinformatic analysis revealed that one of the biological pathways upregulated by B2AR activation in BMDMs was triglyceride biosynthesis (Figure 2C). Biased analysis of top DEGs revealed two candidate genes with known roles in triglyceride biosynthesis in macrophages—*Hilpda* (#25 top DEG at 1‐hour timepoint, Figure 2A and Table S1) and *Dgat1* (#32 top DEG at 4‐hour timepoint, Figure 2B and Table S2). Of note, the *Dgat1* gene contains a CRE sequence in its promoter and *Dgat1* has been validated as a transcriptional target of CREB in myocytes.^42^

We verified our findings by qPCR in multiple ways. Firstly, a time‐course of fenoterol stimulation revealed that *Hilpda* was a downstream target of B2AR activation. *Hilpda* expression peaked at 10‐fold over baseline at 90 minutes post stimulation (Figure 2D). *Dgat1* also responded to B2AR activation with a 10‐fold increase in expression observed at 2‐4 hours post B2AR activation (Figure 2D). Furthermore, NE stimulation increased the expression of *Hilpda* and *Dgat1* in WT BMDMs in a dose‐dependent manner, and this effect was lost in *Adrb2*‐knockout macrophages (Figure 2E). Finally, *Hilpda* and *Dgat1* transcriptional activation by NE was completely abolished by B2AR‐specific antagonist ICI‐118,511 (Figure 2F). Overall, our data were consistent with B2AR driving a lipid storage program in BMDMs. We next set out to determine the functional relevance of increased *Dgat1* and *Hilpda* gene expression.

### 3.3 B2AR stimulation promotes the uptake and storage of extracellular oleate in triglycerides in BMDMs

**FIGURE 3:**
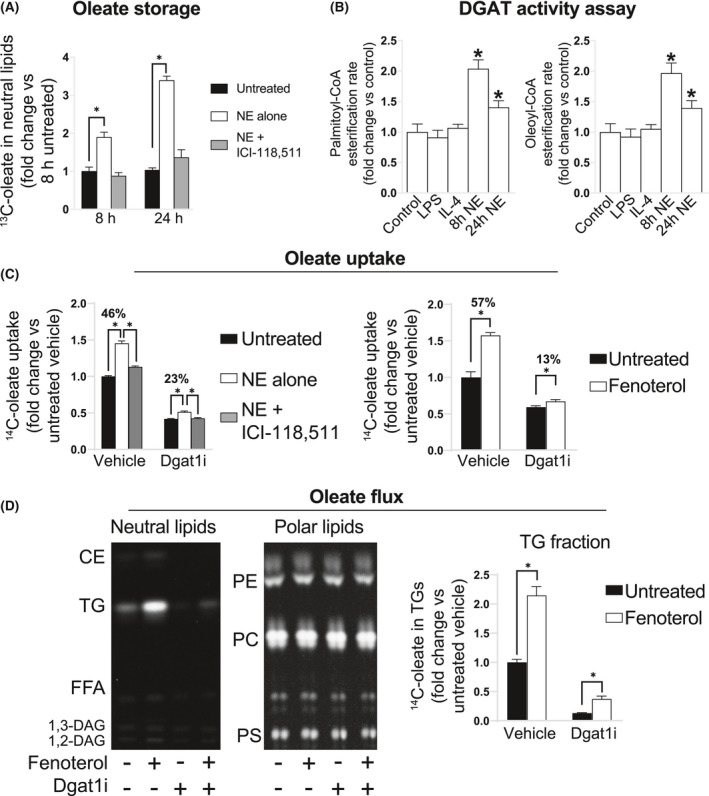
B2AR activation promotes extracellular oleate storage as triglycerides in macrophages in a DGAT1‐dependent manner. A, GC‐MS quantification of ^13^C‐oleate in the neutral lipid fraction of BMDMs stimulated with 1 μM NE in the presence or absence of 1 μM B2AR antagonist ICI‐118,511 and treated with 250 μM ^13^C‐oleate for 8 and 24 h. Values are normalized to the ^13^C‐oleate amount in the neutral lipid fraction of unstimulated cells after 8 h of oleate loading. B, DGAT activity in the microsomal fraction of BMDMs stimulated with 100 ng/mL LPS or 10 μM IL‐4 for 24 h, or 1 μM NE for 8 or 24 h, using palmitoyl‐CoA or oleoyl‐CoA as a substrate, expressed as fold change from untreated cells. C, Oleate uptake by BMDMs, stimulated with 1 μM fenoterol, 1 μM NE, 1 μM B2AR antagonist ICI‐118,511, 1 μM DGAT1 inhibitor A922500, as indicated on the graph, or treated with 250 μM oleate in the presence of ^14^C‐oleate tracer for 16 h. Oleate uptake is expressed as a fold change in specific activity of stimulated cells from unstimulated controls. D, Representative areas of thin‐layer chromatography plate autoradiograms of separated neutral or polar lipids (from the same total lipid samples) isolated from BMDMs stimulated with 1 μM fenoterol and 1 μM A922500 as indicated, then treated with 100 μM oleate containing ^14^C‐oleate tracer for 16 h. Graph shows the quantification of TG band intensities, normalized to the intensity of untreated vehicle group. All graphs show means ± SEM. N = 4 mice in all experiments. In A, C, D, *indicates *P* < .05 compared between treatments using 2‐way ANOVA (in B, 1‐way ANOVA) with Bonferroni's multiple comparisons test

Initially, we investigated whether B2AR activation increased triglyceride biosynthesis in BMDMs. To do so, we stimulated BMDMs with NE and incubated them with stably labeled monounsaturated fatty acid oleate. Oleate is known to be more readily directed toward triglyceride storage by cultured cells when compared to saturated fatty acids such as palmitate.^43^ Analysis of neutral lipids revealed that cells stored similar amounts of ^13^C‐oleate after 8 and 24 hours of incubation, suggesting that BMDMs reached their triglyceride storage capacity limit within the first 8 hours of oleate treatment (Figure 3A). Strikingly, NE enhanced the amount of ^13^C‐oleate stored in neutral lipids by 2‐fold at 8 hours and by 3‐fold at 24 hours of treatment, and this effect could be blocked by ICI‐118,511, indicating that B2AR activation increased triglyceride storage in BMDMs (Figure 3A). In line with these findings, NE treatment increased DGAT activity in BMDM microsomes both at 8 and 24 hours post B2AR activation (Figure 3B). Interestingly, increased DGAT activity was specific to NE, as neither pro‐inflammatory activation by LPS nor anti‐inflammatory activation by interleukin‐4 (IL‐4)‐modulated microsomal DGAT activity in BMDMs (Figure 3B). Of note, DGAT activity in BMDM microsomes in vitro could be completely blocked in the presence of DGAT1‐specific inhibitor A922500 (DGAT1i), indicating that DGAT1 is the predominant DGAT isoform in BMDMs (Figure S2).

We then addressed the importance of increased DGAT1 activity on the uptake and storage of oleate in BMDMs. DGAT1i treatment reduced the total oleate uptake by BMDMs by 2‐fold over 16 hours of incubation, suggesting that fatty acid esterification to triglycerides is one of the main processes driving extracellular fatty acid influx to BMDMs (Figure 3C). Similarly, B2AR activation increased the total oleate uptake by 1.5‐fold, and this increase was markedly diminished in the presence of DGAT1i (Figure 3C). Furthermore, tracing the flux of radiolabel revealed that while oleate was readily incorporated in triglycerides and all major phospholipid classes in BMDMs, B2AR selectively increased oleate flux to triglycerides without affecting the radiolabeling of other lipid species, and inhibiting DGAT1 activity blocked most of the triglyceride storage in BMDMs (Figure 3D). Altogether, our results showed that B2AR activation elevated DGAT1 activity, which increased oleate uptake and storage as triglycerides in BMDMs.

### 3.4 B2AR stimulation reduces the rate of triglyceride lipolysis by HILPDA‐dependent inhibition of ATGL activity in BMDMs

Next, we sought to address the role of B2AR‐mediated *Hilpda* transcriptional activation in promoting triglyceride storage in BMDMs. Given that HILPDA is an endogenous ATGL inhibitor,^33^ we designed a lipolysis assay that allowed us to assess the contribution of different lipolytic pathways to FFA release in BMDMs. We first loaded BMDMs overnight with oleate (containing radiolabeled tracer), then washed the cells, changed the medium, immediately stimulated the cells with B2AR agonists, and monitored the release of radiolabel into the fresh medium. B2AR activation resulted in a reduced rate of intracellular oleate release into the medium, and this could be reversed by B2AR antagonism (Figure 4A).

**IGURE 4:**
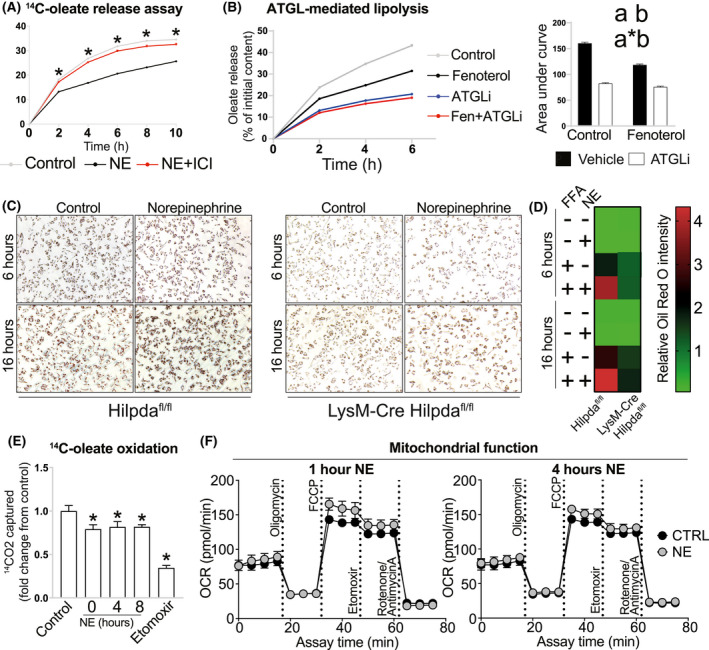
FB2AR activation reduces the rate of oleate release from triglyceride‐laden macrophages by HILPDA‐dependent ATGL inhibition. A, Fractional oleate release into the medium by BMDMs, preloaded with 250 μM oleate in the presence of ^14^C‐oleate tracer for 16 h, at indicated timepoints post medium change. Cells were pretreated with inhibitors 30 min before medium change (inhibitor concentration in the medium was maintained post change) and treated with B2AR agonists immediately after medium change, as indicated on the graphs: A) 1 μM NE, 1 μM B2AR antagonist ICI‐118,511; B) 1 μM fenoterol; 20 μM ATGL inhibitor Atglistatin. C, Representative Oil Red O images of *Hilpda* ^fl/fl^ and Lyz2‐Cre *Hilpda* ^fl/fl^ BMDMs (n = 3/group), treated with 600 μM palmitate:oleate mixture for 6 or 16 h, in the presence or absence of 10 μM NE. D, Quantification of Oil Red O staining intensity. E, Oleate oxidation, assessed over 3 h in cells pretreated with 1 μM NE for indicated periods of time, or in the presence of 40 μM mitochondrial fatty acid oxidation inhibitor etomoxir. F, Mitochondrial stress Seahorse assays of BMDMs treated with 1 μM NE for 1 or 4 h prior to the assay. All graphs show means ± SEM. N = 4 mice in all WT experiments. In A, *indicates *P* < .05 compared between B2AR agonist treatments using two‐way ANOVA with Bonferroni's multiple comparisons test. In B) area under curve graphs, a indicates *P* < .05 for B2AR agonist effect factor, b indicates *P* < .05 for inhibitor effect factor, and a*b indicates *P* < .05 for interaction between both factors in two‐way ANOVA. In E, *indicates *P* < .05 compared between treatments and controls using one‐way ANOVA

We then investigated whether DGAT1‐mediated FFA re‐esterification, or autophagic degradation of lipid droplets (lipophagy),^44^ could explain the reduced oleate release into the media following B2AR activation in BMDMs. We found that inhibiting DGAT1 increased, while blocking autophagy with bafilomycin A1 reduced the oleate release rate in BMDMs (Figure S3A and B). However, both inhibitors remained effective in suppressing the lipolysis in B2AR agonist‐treated macrophages (DGAT1i interaction *P* value = .104, Baf A1 interaction *P* value = .316, Figure S3A and B), indicating that B2AR agonists reduced oleate release neither by regulating fatty acid re‐esterification nor lipophagy. Of all compounds tested, we observed the strongest inhibitory effect on BMDM lipolysis using ATGL‐specific inhibitor atglistatin (Figure 4B). Importantly, atglistatin did not inhibit lipolysis further in fenoterol‐treated macrophages, suggesting that B2AR activation decreased the rate of lipolysis in BMDMs by suppressing ATGL activity (Figure 4B, interaction *P* value <.0001).

In order to demonstrate whether HILPDA was responsible for the inhibition of ATGL activity and increased triglyceride retention in B2AR agonist‐treated macrophages, we obtained BMDMs from macrophage‐specific *Hilpda* knockout mice. Similar to the previous reports,^30^, ^34^ we observed a strongly impaired capacity for *Hilpda*‐deficient macrophages to accumulate lipid droplets in the presence of exogenous fatty acids (Figure 4C and D, S4). Importantly, while B2AR activation by NE increased the neutral lipid stores in control BMDMs loaded with fatty acids for both 6 and 16 hours, this increase was largely diminished in macrophages lacking *Hilpda* (Figure 4C and D), indicating that elevated *Hilpda* expression in response to B2AR activation contributes to the increased triglyceride storage in macrophages.

Lastly, we investigated whether the B2AR‐mediated suppression of triglyceride lipolysis also affected fatty acid oxidation in macrophages. Indeed, we observed a small decrease in the oxidation of exogenous oleate in NE‐treated BMDMs (Figure 4E). However, B2AR activation did not affect the mitochondrial respiration in macrophages, suggesting that decreased oleate oxidation in response to NE treatment was not due to changes in mitochondrial function (Figure 4F). Overall, we found that B2AR activation suppressed macrophage ATGL‐dependent lipolysis by upregulating *Hilpda* expression.

### 3.5 B2AR stimulation favors the increase in the synthesis rate of polyunsaturated fatty acid‐containing triglycerides

**FIGURE 5:**
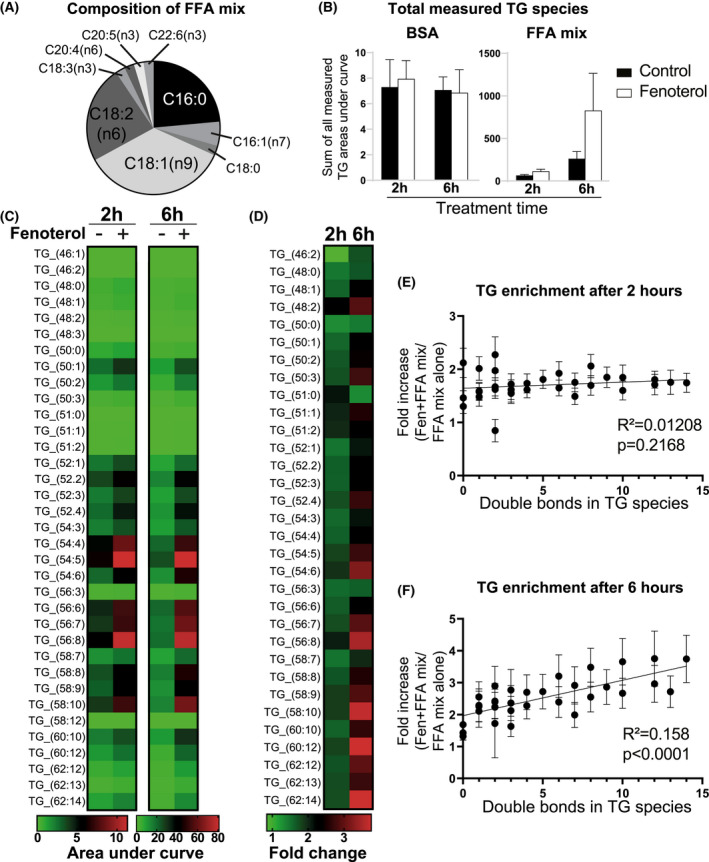
B2AR activation favors the synthesis of PUFA‐rich triglycerides in macrophages during the incubation with a complex FFA mixture. A, Fatty acid composition of the mixture used in the experiment. B, Sum of areas under curve of all triglyceride species measured by LC‐MS in BMDMs treated with 0.5% BSA alone or 500 μM of FFA mixture conjugated to 0.5% BSA for 2 or 6 h, in the presence or absence of 1 μM fenoterol as indicated. C, Heatmap of areas under curve of each measured triglyceride species in BMDMs treated with FFA mixture for 2 or 6 h, in the presence or absence of 1 μM fenoterol as indicated (BSA alone treatment is not shown). D, Fold change in the abundance of each measured triglyceride species between BMDMs treated with 1 μM fenoterol and untreated controls in the presence of FFA mix for 2 or 6 h. E, Linear regression plots of fold change in the abundance of each measured triglyceride species between BMDMs treated with 1 μM fenoterol and untreated controls plotted against the number of double bonds in the respective triglyceride molecule, at either 2 or F) 6 h of incubation. All graphs show means ± SEM. N = 4 mice in all experiments. In B‐D, significant differences were not assessed. In E, F, *R* ^2^ and *P* values of linear regression are indicated on graphs

So far, all our conclusions relied on experiments involving labeled oleate measurements. In order to demonstrate whether B2AR activation increased BMDM triglyceride storage independently of the type of exogenous fatty acid used as a substrate, we formulated a complex FFA mixture that had similar ratios of FFAs released from WAT in response to fasting in vivo (Figure 5A). We then treated BMDMs with fenoterol for 2 and 6 hours in the presence of FFA mixture and performed lipidomic analysis to reveal global changes in BMDM lipid species induced by B2AR activation. Interestingly, we found that fenoterol treatment did not modulate overall triglyceride levels in BMDMs in the absence of FFA treatment (Figure 5B), suggesting that elevated extracellular FFA concentrations are required for the B2AR‐mediated increase in triglyceride storage (note that all treatments were performed in the media supplemented with 10% fetal bovine serum that contains FFAs). Indeed, in line with our previous data obtained using oleate, treatment with FFA mixture increased BMDM triglyceride storage in a time‐dependent manner, which was accelerated further in fenoterol‐treated cells (Figure 5B).

Analysis of individual lipid species revealed that while the treatment with FFA mixture affected the abundance of several phospholipid species, B2AR activation did not modulate the level of any measured PC, PE, or DAG species in BMDMs, either in the presence or absence of FFA mixture (Figure S5). In contrast, fenoterol treatment enhanced the accumulation of nearly all measured triglyceride species in BMDMs treated with FFA mixture at both 2‐ and 6‐hour timepoints (Figure 5C and D). Interestingly, while at 2 hours fenoterol increased the accumulation of most measured triglyceride species in a uniform manner (Figure 5D and E), at 6 hours the increase was disproportionately shifted toward the storage of polyunsaturated fatty acid‐containing triglycerides (Figure 5D,F). In general, we found that B2AR activation increased triglyceride storage in BMDMs only in the presence of elevated extracellular FFA concentrations, that it did not affect the levels of measured phospholipid species, and that it preferentially increased the synthesis rate of polyunsaturated fatty acid‐containing triglycerides.

### 3.6 Macrophages isolated from injured hearts have a transcriptional profile indicative of increased triglyceride storage

**FIGURE 6:**
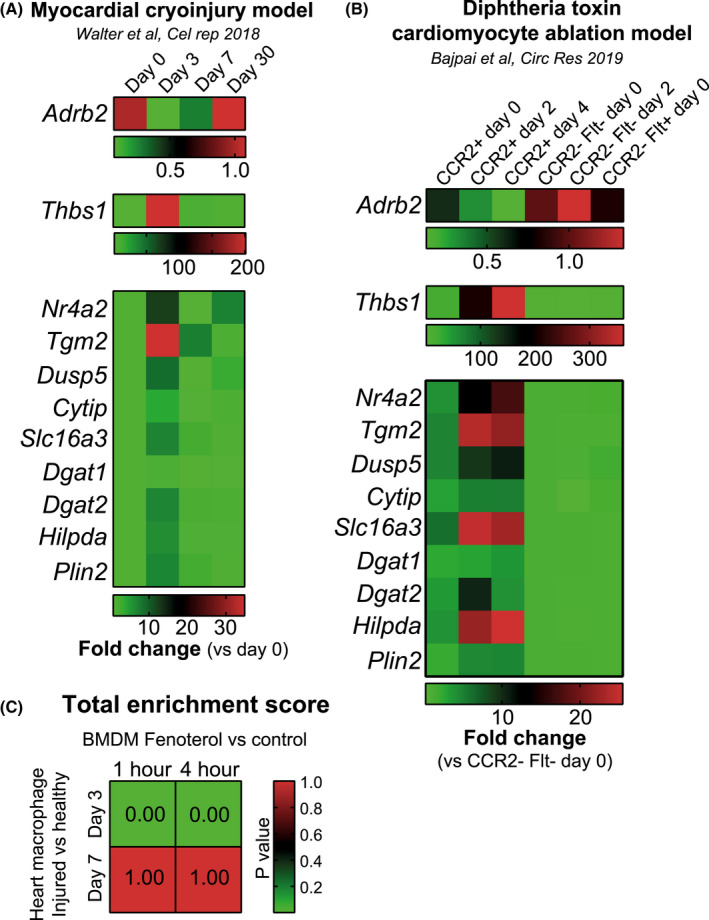
Macrophages isolated from injured hearts have a transcriptional signature indicative of B2AR activation and increased triglyceride storage. A, Heatmaps showing the expression levels of indicated genes measured in macrophages isolated from mice subjected to myocardial cryoinjury model at indicated periods following the injury. Expression levels are normalized to macrophages from healthy hearts (day 0). N = 3 mice/group. Data were obtained from Walter et al B, Heatmaps showing the expression levels of indicated genes measured in indicated macrophage populations isolated from mice with cardiomyocyte‐specific expression of diphtheria toxin receptor at indicated periods following the administration of diphtheria toxin. Expression levels are normalized to CCR2‐Flt‐macrophages from healthy hearts (day 0). N = 2‐4 mice/group. Experimental data generated by Bajpai et al were obtained from Immgen.com. C, Statistical comparison of global transcriptomic changes induced by fenoterol stimulation in BMDMs in vitro (this study, normalized to unstimulated controls) to changes induced by myocardial cryoinjury in vivo (Walter et al, normalized to Day 0 macrophages). Lower *P* value indicates higher similarities of changes between two datasets. In A, B, statistical differences were not assessed and multiple heatmaps were generated to improve the visualization of the data

Finally, we sought to understand whether B2AR activation‐mediated transcriptional changes in triglyceride biosynthesis genes are observed in macrophages in vivo. We chose to investigate macrophages isolated from injured hearts for the following reasons: (i) Heart injury increases sympathetic nerve activation in the heart, leading to elevated NE levels^35^; (ii) Macrophage B2ARs are necessary for survival post injury,^11^ indicating that B2AR‐dependent processes in macrophages are required for heart repair; (iii) The transcriptomic analyses of heart macrophages isolated from multiple different mouse models of heart injury are available^45^, ^46^, ^47^, ^48^; (iv) Recent single‐cell RNAseq analysis revealed that ‘lipid transport’ was the most upregulated pathway in heart macrophages in response to myocardial infarction.^47^ We found that both myocardial cryoinjury^45^ and diphtheria toxin‐mediated ablation of cardiomyocytes^46^ resulted in downregulated *Adrb2* levels in heart macrophages at 2‐4 days post injury (Figure 6A and B), similar to reduced *Adrb2* expression in response to NE in BMDMs (Figure 1A). We then analyzed the expression of our top RNAseq DEGs (Figure 2A and B, Tables S1 and S2) in both heart macrophage datasets. We observed that all genes that were highly upregulated in response to fenoterol stimulation in BMDMs were also increased in heart macrophages acutely after heart injury (Figure 6A and B). Importantly, the expression of triglyceride storage markers *Dgat1*, *Dgat2* (DGAT isoform that has high gene expression levels in heart macrophages, relative to other tissue macrophages, according to Immgen.com), *Hilpda,* and *Plin2* (encoding Adipose differentiation‐related protein, a marker for cellular lipid droplet accumulation^49^) was also increased in cardiac macrophages in response to injury (Figure 6A and B), indicating that heart injury in vivo drives a similar triglyceride storage program in macrophages as NE stimulation in vitro. Finally, statistical comparison of global transcriptomic changes induced by NE in vitro to heart injury in vivo revealed a high similarity between the B2AR activation in BMDMs and heart macrophages at 3 days post injury, which was no longer observed at 7 days post injury (Figure 6C). Altogether, we found that myocardial injury, associated with elevated sympathetic nervous system activity in the heart, increased the transcription of genes involved in triglyceride biosynthesis in macrophages, suggesting that our described NE‐B2AR pathway could also increase triglyceride storage in macrophages in vivo.

## 4 DISCUSSION

Here, we demonstrate that the sympathetic neurotransmitter NE increases triglyceride storage in mouse BMDMs by activating B2AR. We show that these effects are mediated by the upregulated expression of two genes: *Dgat1*, leading to increased triglyceride biosynthesis; and *Hilpda*, leading to a partial ATGL inhibition and decreased triglyceride lipolysis. Our study is the first to link NE‐B2AR signaling to changes in macrophage lipid metabolism, namely, to the increased triglyceride storage.

Previous studies have shown that pro‐inflammatory activation leads to triglyceride accumulation in macrophages.^22^, ^23^, ^24^, ^25^, ^26^, ^27^ The precise mechanisms leading up to increased triglyceride storage following the stimulation of macrophages with bacterial components have not been fully elucidated. However, it has been suggested that decreased lipolysis,^23^, ^25^ decreased fatty acid oxidation,^27^ or a reduction in extracellular pH^24^ could be involved. Importantly, pro‐inflammatory macrophage activation is accompanied by dramatic changes in macrophage metabolism, including increased glycolysis and phospholipid biosynthesis, and decreased fatty acid oxidation.^50^ In contrast, our data indicate that B2AR activation in BMDMs specifically increases triglyceride storage only in the presence of elevated extracellular fatty acid levels, and without affecting either the levels or the fatty acid composition of other cellular lipid species. Furthermore, while B2AR activation partially suppressed exogenous fatty acid oxidation, it did not inhibit mitochondrial oxidative metabolism, which is a common feature of pro‐inflammatory macrophage activation.^51^ The decrease in exogenous oleate oxidation following B2AR stimulation was consistent with an increased fatty acid flux toward lipid droplets and away from mitochondria. Lastly, we find that DGAT activity is not affected by macrophage inflammatory activation (Figure 3B), suggesting that the mechanisms leading to triglyceride storage in response to B2AR activation in macrophages are mechanistically distinct from those observed in response to stimulation with bacterial components. Therefore, to our knowledge, NE‐B2AR signaling axis is the first reported pathway that specifically modulates macrophage triglyceride metabolism.

We have also found that B2AR activation preferentially increased the storage of polyunsaturated fatty acid‐containing triglycerides. While we do not provide a mechanistic explanation for this observation, a recent study has found that increased HILPDA expression enriches cancer cells in polyunsaturated fatty acid‐containing triglycerides.^52^ Therefore, it is plausible that it is the increased HILPDA expression that mediates our observed phenotype in macrophages. In future, it would be of interest to investigate the precise molecular mechanism of how HILPDA modulates the profile of cellular triglyceride species.

The main limitation of our study is the lack of direct experimental evidence that NE‐B2AR is involved in regulating triglyceride metabolism in macrophages in vivo. However, there are multiple physiological and disease settings where increased tissue sympathetic nerve activity coincides with the accumulation of lipid droplets in tissue macrophages. During aging, sympathetic nerve activity is increased in the brain, as evidenced by higher brain NE turnover.^53^ The aging brain is also characterized by the presence of lipid droplet‐containing microglia, which have been shown to promote neurodegeneration.^19^ Interestingly, triglyceride‐laden microglia isolated from aging brains also exhibits downregulated *Adrb2* expression,^19^ which can be a marker of increased B2AR activation, as we have shown here (Figure 1A). Furthermore, fasting promotes sympathetic nerve firing in white adipose tissues, which leads to adipocyte lipolysis, but lipid droplet accumulation in adipose tissue macrophages.^14^, ^15^ Finally, our analyses suggest that heart macrophages accumulate triglycerides in response to heart injury (Figure 6), which is also characterized by increased sympathetic nerve activity.^35^ Two recent single‐cell RNAseq analyses also show similar findings—one revealed a population of lipid‐associated macrophages in the heart post injury,^48^ while the other demonstrated that myocardial infarction upregulates lipid transport pathways in cardiac macrophages.^47^ While the involvement of B2AR in these processes is currently speculative, we believe that there is a firm basis to investigate the relationship between B2AR activation and lipid‐laden macrophage formation in vivo in future using cell type‐specific *Adrb2* KO mice.

We also did not address the biological importance of the NE signaling‐dependent increase in macrophage triglyceride storage. However, we speculate that it might have different physiological outcomes, depending on the context. For example, heart tissue is enriched in polyunsaturated fatty acid‐containing phospholipids.^54^ Furthermore, heart injury is characterized by elevated reactive oxygen species (ROS).^55^ As polyunsaturated fatty acids are selectively released from apoptotic cells,^56^ and ROS can rapidly oxidize them, sequestering polyunsaturated fatty acids within lipid droplets may protect them from peroxidation and subsequent lipotoxicity.^57^ Intriguingly, systemic atglistatin treatment has already been shown to improve the recovery from heart failure in mice.^58^ While these effects were originally attributed to inhibited adipose tissue lipolysis,^35^, ^58^ it is tempting to speculate that atglistatin‐mediated increase in triglyceride storage in macrophages could also be beneficial for cardiac repair. In future, it would be interesting to investigate such hypothesis by subjecting macrophage‐specific *Dgat1* or *Hilpda* knockout mice (macrophages from both models exhibit defects in triglyceride storage) to myocardial injury, and assessing their recovery.

Alternatively, adipose tissue macrophages have been suggested to buffer local fatty acid levels released from adipocytes during lipolysis.^14^, ^59^ It is therefore plausible that NE can simultaneously promote fatty acid release from adipocytes and fatty acid storage in adipose tissue macrophages, ensuring an efficient lipolytic response without subjecting white adipose tissue‐resident cells to lipotoxic FFA concentrations. Furthermore, a preference toward the storage of essential polyunsaturated fatty acids in adipose tissue macrophages might be a physiological mechanism that lowers the polyunsaturated fatty acid efflux from the adipose tissue during fasting, limit their oxidation in other tissues, and thus preserve systemic stores. Overall, future studies are warranted to investigate the importance of NE‐B2AR signaling in various physiological and pathological settings.

Finally, it is likely that the B2AR pathway we describe here is involved in regulating triglyceride storage in other cell types as well. B2AR agonists increase hepatic lipid accumulation in mice through a mechanism that is independent of white adipose tissue lipolysis.^60^ Age‐related hepatic triglyceride accumulation is attenuated in *Adrb2* knockout animals,^61^ while overexpressing *Adrb2* in hepatocytes promotes hepatosteatosis.^60^ Furthermore, NE released during intensive exercise regulates myofiber remodeling in skeletal muscle, a process associated with a CREB‐dependent increase in DGAT1 activity and elevated storage of intramuscular triglycerides.^42^ Lastly, while NE released during fasting activates lipolysis in white adipocytes, it also increases their DGAT1 activity and fatty acid re‐esterification, which has been reported to protect adipocytes from endoplasmic reticulum and lipotoxic stresses.^62^ Even though NE is classically viewed as a catabolic neurotransmitter, we believe that it also has an anabolic role in many cell types, including macrophages.

## DISCLOSURES

K. Petkevicius is presently employed by AstraZeneca. No other conflicts of interest in connection with this article are declared.

## AUTHOR CONTRIBUTIONS

K. Petkevicius, S. Virtue, and A. Vidal‐Puig conceptualized the study. K. Petkevicius, G. Bidault, and S. Virtue performed research. B. Jenkins and A. Koulman performed LC‐MS lipid analysis. XAMH van Dierendonck and R. Stienstra performed experiments on *Hilpda* KO macrophages. A. Dugourd and J. Saez‐Rodriguez analyzed transcriptomic data. S. Virtue and A. Vidal‐Puig supervised the study. K. Petkevicius wrote the study.

## Supporting information

Fig S1

Fig S2

Fig S3

Fig S4

Fig S5

Table S1

Table S2

## Data Availability

NGS data in this manuscript have been deposited in GEO, accession number GSE160640.
