# Supplementary material for: Norepinephrine promotes triglyceride storage in macrophages via beta2‐adrenergic receptor activation
Source: FASEB J. 2021 Jan 23;35(2):e21266. doi: 10.1096/fj.202001101R (PMC7898725; doi:10.1096/fj.202001101R)
Supplement: Supplementary file 1 — Fig S1 [file FSB2-35-e21266-s006.docx]

**Supplementary figure 1**


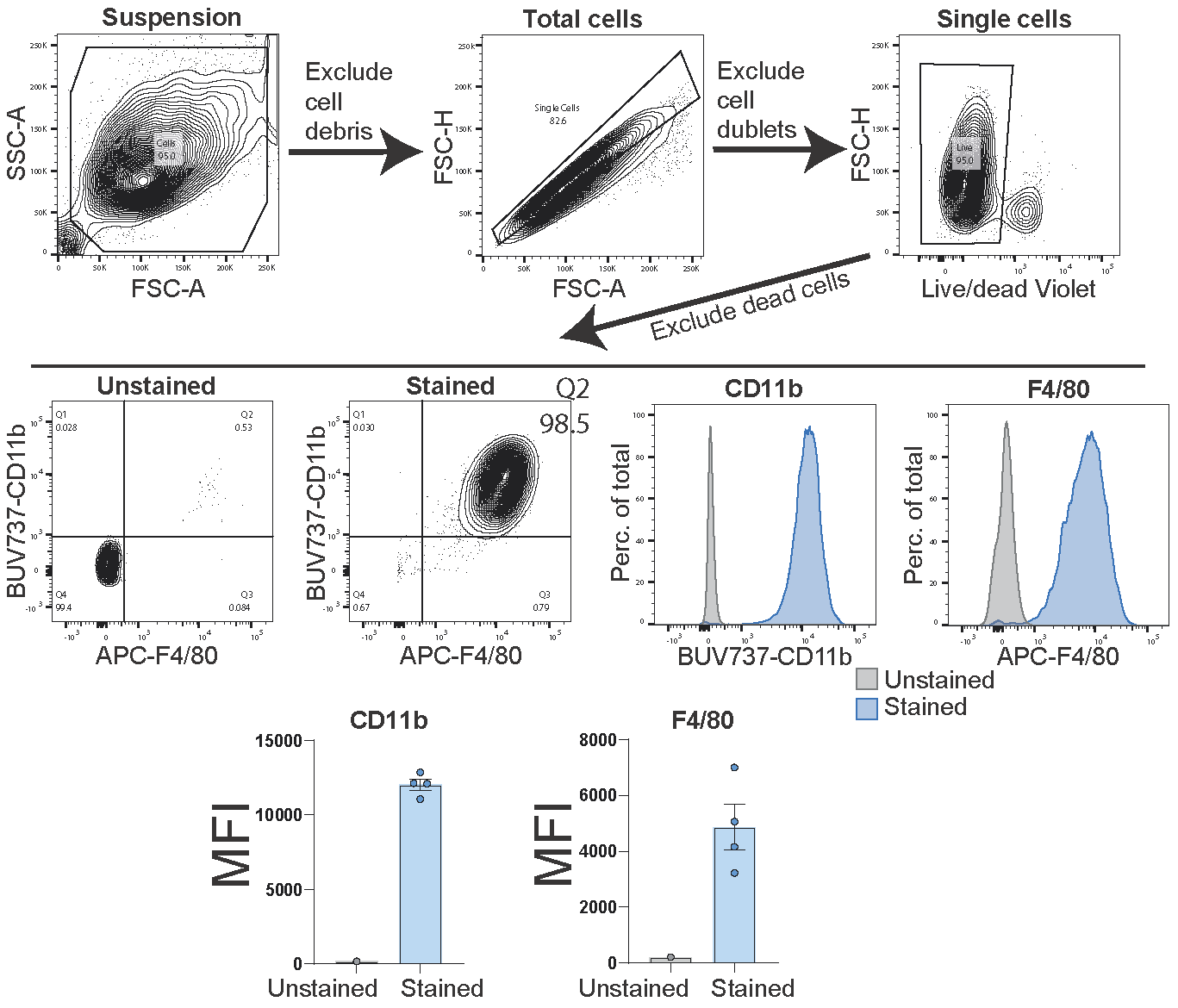


**Supplementary figure 1.** Flow cytometry analysis of a representative bone marrow-derived macrophage culture. Panels indicate a sequential gating strategy to obtain single live cells, which are either unstained or stained with antibodies against macrophage surface markers CD11b and F4/80. Analysis indicate a high percentage (>98%) of bone marrow-derived cells expressing macrophage surface markers following 7 days of differentiation.
