# Supplementary material for: Norepinephrine promotes triglyceride storage in macrophages via beta2‐adrenergic receptor activation
Source: FASEB J. 2021 Jan 23;35(2):e21266. doi: 10.1096/fj.202001101R (PMC7898725; doi:10.1096/fj.202001101R)
Supplement: Supplementary file 2 — Fig S2 [file FSB2-35-e21266-s005.docx]

**Supplementary figure 2**


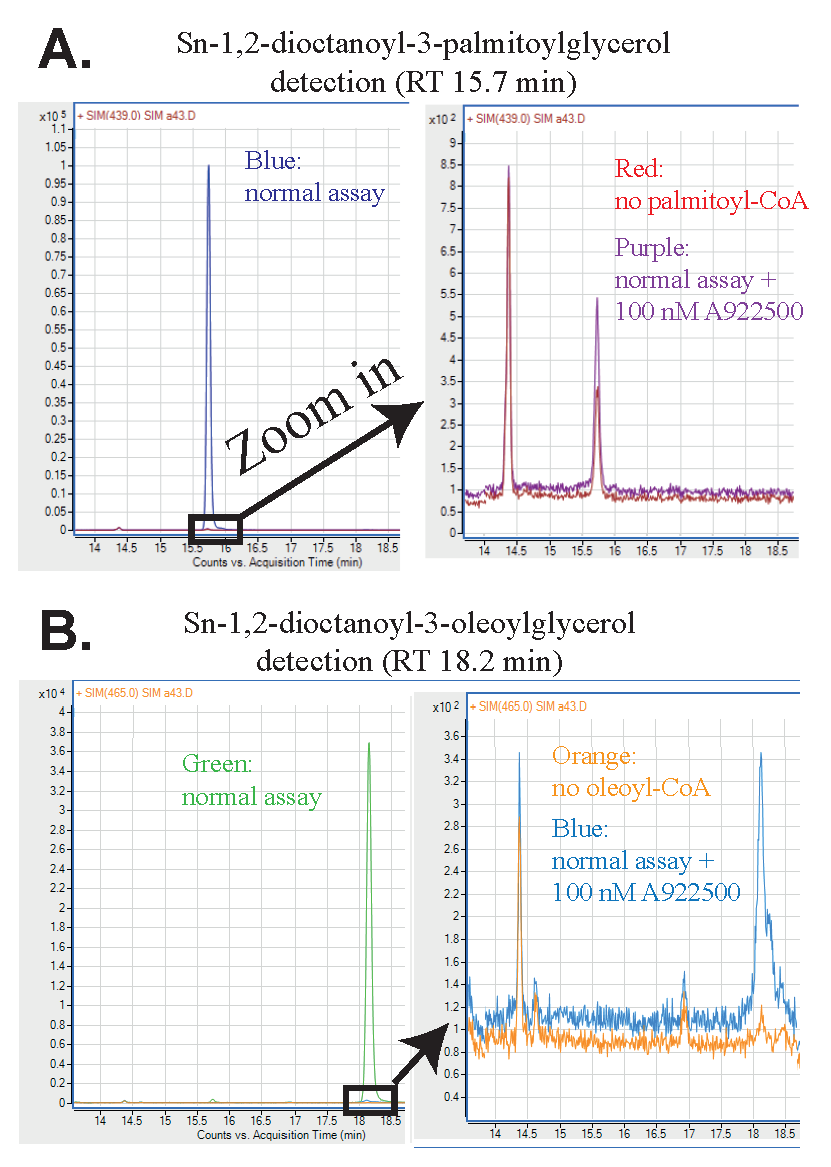


**Supplementary figure 2.** Fragments of GC-MS chromatograms of three DGAT activity assays, performed on the same microsomal fraction isolated from untreated BMDMs. Assays were either performed normally, or in the presence of 100nM DGAT1 inhibitor A922500, or without fatty acyl-CoA in the reaction, as indicated on the graphs. Note that data in the panels A and B were obtained in the same GC-MS run in a specific ion monitoring mode, and presented for different ions: A) 439.0 m/z and B) 465.0 m/z. Also note the same acquisition time axis scale but difference in counts axis scale between left and right panels.
