# Supplementary material for: Norepinephrine promotes triglyceride storage in macrophages via beta2‐adrenergic receptor activation
Source: FASEB J. 2021 Jan 23;35(2):e21266. doi: 10.1096/fj.202001101R (PMC7898725; doi:10.1096/fj.202001101R)
Supplement: Supplementary file 3 — Fig S3 [file FSB2-35-e21266-s004.docx]

**Supplementary figure 3**


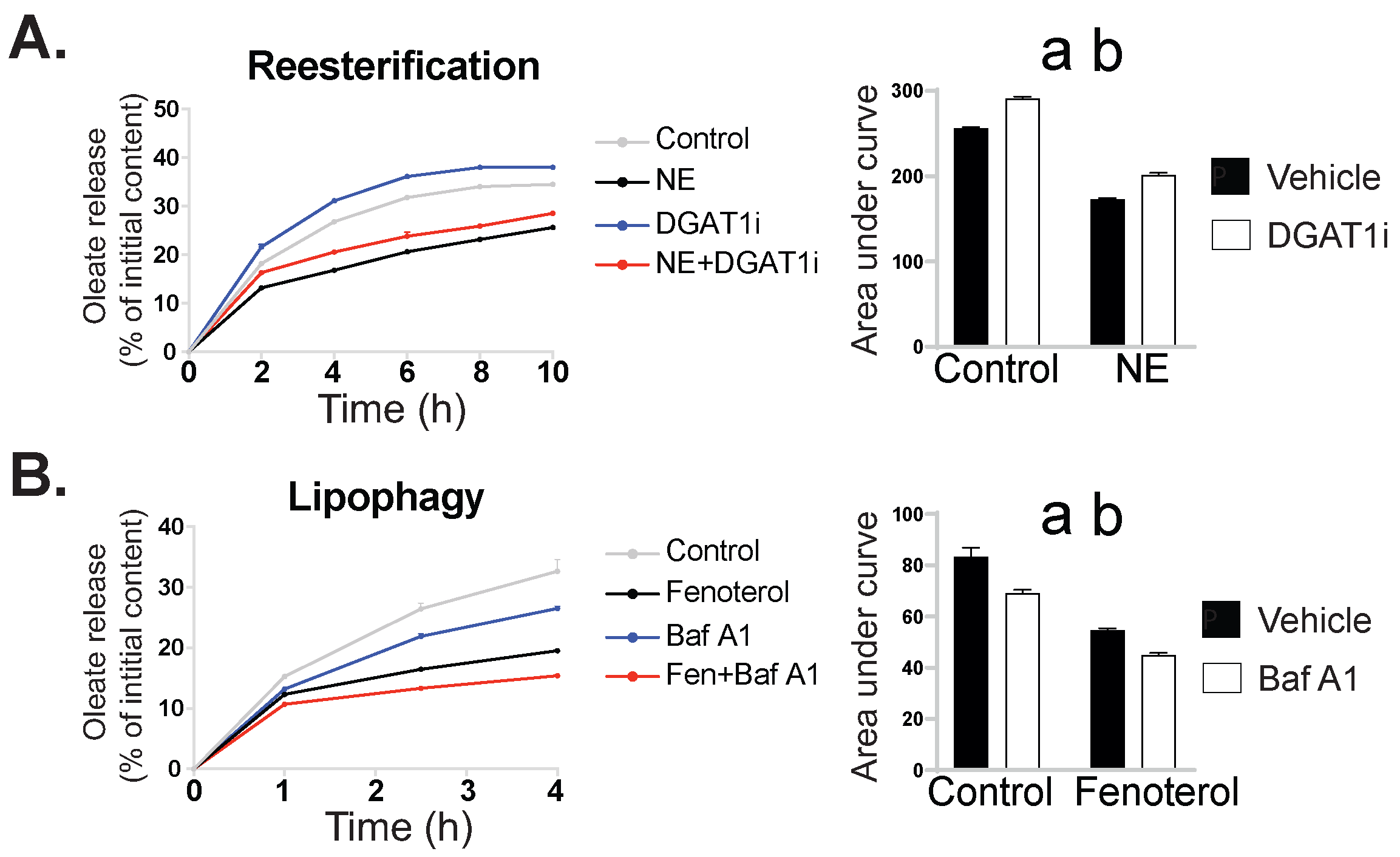


**Supplementary figure 3.** Fractional oleate release into the medium by BMDMs, pre-loaded with 250 μM oleate in the presence of ^14^C-oleate tracer for 16 h, at indicated time-points post medium change. Cells were pre-treated with inhibitors 30 min before medium change (inhibitor concentration in the medium was maintained post change) and treated with B2AR agonists immediately after medium change, as indicated on the graphs: A) 1 μM NE, 1 μM DGAT1 inhibitor A922500; B) 1 μM fenoterol, 100 nM bafilomycin A1. All graphs show means ± SEM. N=4 mice in all experiments. In area under curve graphs, a indicates p < 0.05 for B2AR agonist effect factor, b indicates p < 0.05 for inhibitor effect factor and a*b indicates p < 0.05 for interaction between both factors in two-way ANOVA.
