# Supplementary material for: Norepinephrine promotes triglyceride storage in macrophages via beta2‐adrenergic receptor activation
Source: FASEB J. 2021 Jan 23;35(2):e21266. doi: 10.1096/fj.202001101R (PMC7898725; doi:10.1096/fj.202001101R)
Supplement: Supplementary file 4 — Fig S4 [file FSB2-35-e21266-s007.docx]

**Supplementary figure 4**


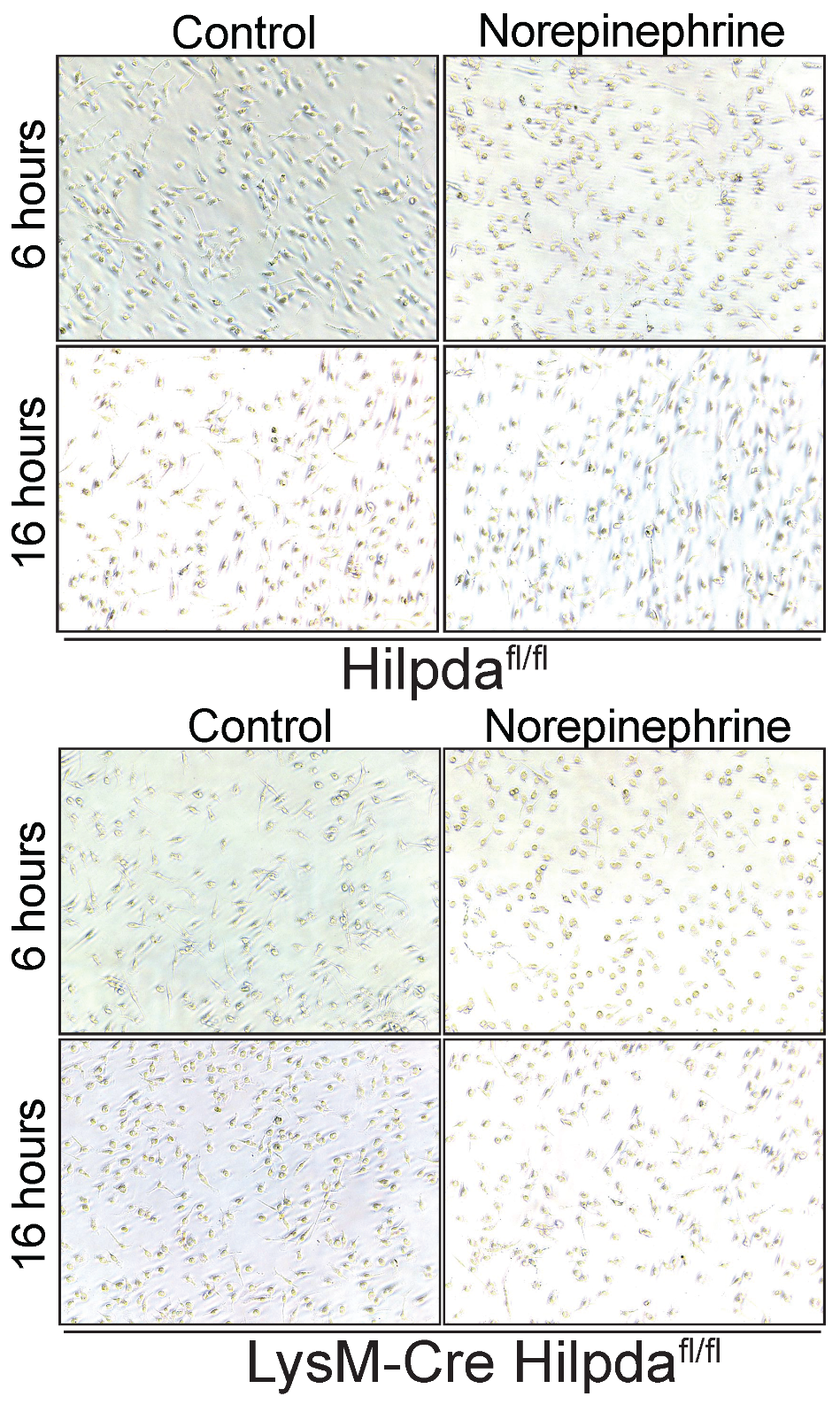


**Supplementary figure 4.** Representative control Oil Red O images of *Hilpda*^fl/fl^ and Lyz2-Cre *Hilpda*^fl/fl^ BMDMs (n=3/group), treated with BSA for 6 or 16 hours, in the presence or absence of 10 μM NE. Images were controls for the experiment presented in Fig. 4C-D.
