# Supplementary material for: Norepinephrine promotes triglyceride storage in macrophages via beta2‐adrenergic receptor activation
Source: FASEB J. 2021 Jan 23;35(2):e21266. doi: 10.1096/fj.202001101R (PMC7898725; doi:10.1096/fj.202001101R)
Supplement: Supplementary file 5 — Fig S5 [file FSB2-35-e21266-s003.docx]

**Supplementary figure 5**


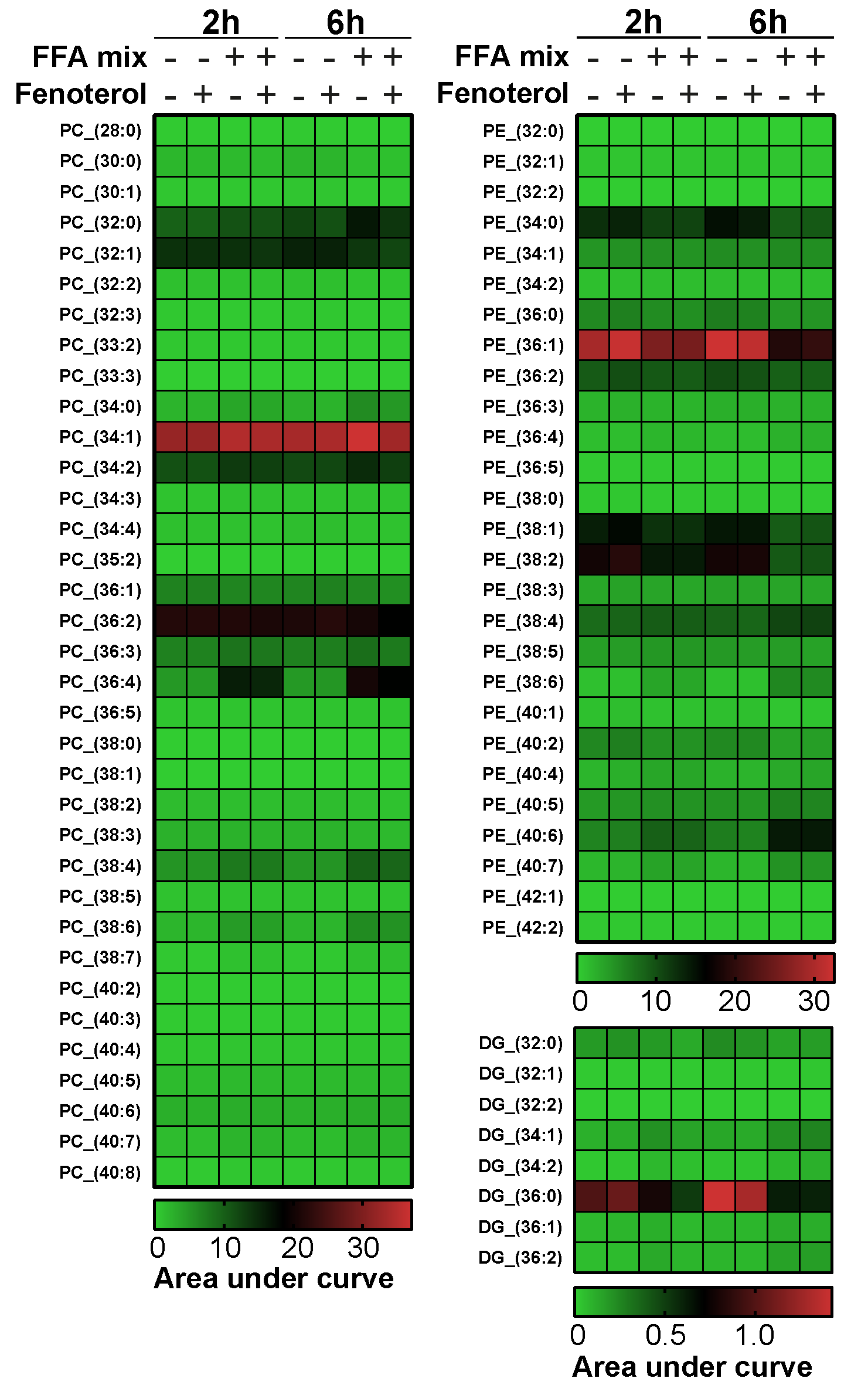


**Supplementary figure 5.** Heatmap of areas under curve of each measured phosphatidylcholine, phosphatidylethanolamine and diacylglycerol species in BMDMs treated with FFA mixture for 2 or 6 hours, in the presence or absence of 1 μM fenoterol as indicated. N=4 mice. Significant differences were not assessed.
