# Supplementary material for: Norepinephrine promotes triglyceride storage in macrophages via beta2‐adrenergic receptor activation
Source: FASEB J. 2021 Jan 23;35(2):e21266. doi: 10.1096/fj.202001101R (PMC7898725; doi:10.1096/fj.202001101R)
Supplement: Supplementary file 6 — Table S1 [file FSB2-35-e21266-s002.docx]

**Supplementary table 1.** Top 100 differentially expressed genes between BMDMs treated with 1 μM fenoterol for 1 hour and untreated controls, ranked based on adjusted p value of change.

| **Gene name** | **Log (Fold change)** | **Average expression** | **p value** | **Adjusted p value** |
| --- | --- | --- | --- | --- |
| FOSL2 | 5.424779 | 12.29506 | 5.03E-19 | 6.99E-15 |
| FOSB | 7.772025 | 8.979583 | 2.02E-18 | 1.40E-14 |
| NR4A2 | 6.601601 | 9.04992 | 4.69E-18 | 2.17E-14 |
| DUSP5 | 5.997088 | 9.893106 | 1.01E-17 | 2.86E-14 |
| THBS1 | 6.24379 | 15.37731 | 1.03E-17 | 2.86E-14 |
| B4GALT5 | 2.380816 | 12.72327 | 1.42E-17 | 2.87E-14 |
| CYTIP | 4.190889 | 11.87469 | 1.45E-17 | 2.87E-14 |
| JDP2 | 3.831992 | 9.418363 | 6.99E-17 | 1.21E-13 |
| CBARP | 3.577609 | 7.85805 | 4.00E-16 | 6.18E-13 |
| ETS2 | 3.314338 | 11.08148 | 4.50E-16 | 6.25E-13 |
| RRP1B | 2.807398 | 9.088756 | 6.10E-16 | 7.70E-13 |
| PLAUR | 2.925662 | 11.35404 | 7.86E-16 | 8.59E-13 |
| PER1 | 2.533183 | 10.53963 | 8.03E-16 | 8.59E-13 |
| SLC25A33 | 4.347321 | 8.666928 | 9.13E-16 | 9.07E-13 |
| DUSP1 | 4.060302 | 10.4049 | 1.06E-15 | 9.83E-13 |
| FOS | 4.066386 | 12.09094 | 1.25E-15 | 1.08E-12 |
| OSM | 3.480799 | 9.296355 | 2.47E-15 | 1.87E-12 |
| MIDN | 2.153464 | 10.64711 | 2.77E-15 | 1.87E-12 |
| PIM1 | 2.701017 | 11.10867 | 2.78E-15 | 1.87E-12 |
| TGM2 | 2.85874 | 12.97633 | 2.79E-15 | 1.87E-12 |
| ID3 | 3.729169 | 8.941129 | 2.83E-15 | 1.87E-12 |
| CFAP43 | 2.331726 | 10.23769 | 3.20E-15 | 2.02E-12 |
| NFIL3 | 4.278378 | 10.75188 | 4.89E-15 | 2.96E-12 |
| THAP6 | 3.296521 | 9.91523 | 6.03E-15 | 3.49E-12 |
| **HILPDA** | 3.350678 | 9.186574 | 6.86E-15 | 3.82E-12 |
| CREM | 4.708477 | 11.14653 | 9.42E-15 | 5.03E-12 |
| CASS4 | 3.67797 | 10.45168 | 1.07E-14 | 5.51E-12 |
| THBD | 2.998192 | 9.391472 | 1.18E-14 | 5.51E-12 |
| CYTH1 | 2.107909 | 12.22054 | 1.22E-14 | 5.51E-12 |
| CCRL2 | 2.910886 | 10.13819 | 1.23E-14 | 5.51E-12 |
| NDEL1 | 2.335768 | 11.30722 | 1.26E-14 | 5.51E-12 |
| PDE4B | 3.325541 | 11.13317 | 1.27E-14 | 5.51E-12 |
| KLF4 | 3.449354 | 9.155293 | 1.39E-14 | 5.85E-12 |
| MXI1 | 2.122942 | 11.60536 | 1.44E-14 | 5.88E-12 |
| IER2 | 2.186608 | 9.248875 | 1.68E-14 | 6.65E-12 |
| GOT1 | 2.381726 | 12.05794 | 1.74E-14 | 6.65E-12 |
| NR4A3 | 5.265912 | 7.380011 | 1.77E-14 | 6.65E-12 |
| SPSB1 | 3.054126 | 7.490097 | 1.86E-14 | 6.80E-12 |
| NUAK2 | 1.923714 | 10.58158 | 2.33E-14 | 8.32E-12 |
| SPTY2D1 | 2.896411 | 10.75676 | 2.63E-14 | 9.07E-12 |
| GCSH | 2.440572 | 10.77913 | 2.67E-14 | 9.07E-12 |
| ADORA2B | 3.975182 | 8.306417 | 4.40E-14 | 1.45E-11 |
| DUSP7 | -2.39575 | 8.176077 | 4.69E-14 | 1.52E-11 |
| CD14 | 2.383338 | 15.20939 | 5.20E-14 | 1.64E-11 |
| ZCCHC14 | 2.820607 | 9.70357 | 6.13E-14 | 1.89E-11 |
| PXDC1 | 2.679641 | 6.381861 | 7.03E-14 | 2.13E-11 |
| KRAS | 1.678617 | 9.827644 | 7.86E-14 | 2.33E-11 |
| ARID5A | 1.910716 | 9.794907 | 9.79E-14 | 2.84E-11 |
| KCNJ2 | -3.15394 | 7.888283 | 1.08E-13 | 3.00E-11 |
| PVR | 2.55941 | 9.633128 | 1.08E-13 | 3.00E-11 |
| BTG2 | 3.043968 | 10.87032 | 1.11E-13 | 3.03E-11 |
| SLC16A3 | 2.91047 | 11.96858 | 1.13E-13 | 3.03E-11 |
| CITED2 | -1.70325 | 9.029236 | 1.52E-13 | 3.97E-11 |
| NOD2 | 2.373967 | 9.323438 | 1.54E-13 | 3.97E-11 |
| **DGAT1** | 3.081875 | 11.08892 | 1.72E-13 | 4.35E-11 |
| CDKN1A | 2.100456 | 13.38769 | 2.12E-13 | 5.23E-11 |
| PDE4D | 2.444419 | 8.225374 | 2.14E-13 | 5.23E-11 |
| JUNB | 3.442796 | 11.73245 | 2.67E-13 | 6.41E-11 |
| PRUNE1 | 1.795344 | 11.02112 | 2.74E-13 | 6.46E-11 |
| GADD45A | -1.99908 | 8.063997 | 2.94E-13 | 6.82E-11 |
| NR4A1 | 4.876522 | 9.706711 | 3.31E-13 | 7.55E-11 |
| VEGFA | 3.809429 | 12.20177 | 4.07E-13 | 9.12E-11 |
| RAB11FIP1 | 1.763881 | 9.850896 | 5.66E-13 | 1.25E-10 |
| RASSF3 | 1.911878 | 12.1281 | 5.82E-13 | 1.26E-10 |
| ZFP667 | 2.037667 | 7.262823 | 6.68E-13 | 1.43E-10 |
| TNFSF14 | 2.212047 | 8.628541 | 6.99E-13 | 1.45E-10 |
| USP36 | 1.333141 | 9.752385 | 7.00E-13 | 1.45E-10 |
| GAN | 2.498512 | 10.84563 | 7.72E-13 | 1.56E-10 |
| BAMBI | 2.368388 | 7.514116 | 7.73E-13 | 1.56E-10 |
| CEBPB | 2.847903 | 9.265876 | 8.78E-13 | 1.74E-10 |
| MRM1 | 1.729796 | 9.178981 | 8.96E-13 | 1.75E-10 |
| TAF11 | 1.960555 | 10.17123 | 9.79E-13 | 1.89E-10 |
| ZFP703 | 1.702068 | 10.8234 | 1.00E-12 | 1.91E-10 |
| TMEM2 | 2.343631 | 9.71596 | 1.17E-12 | 2.20E-10 |
| TAL1 | 1.431355 | 9.354939 | 1.31E-12 | 2.43E-10 |
| ANKRD33B | 1.793644 | 11.19484 | 1.43E-12 | 2.62E-10 |
| ZC3H12A | 1.462076 | 10.31217 | 1.47E-12 | 2.65E-10 |
| PTGS2 | 4.646334 | 7.678188 | 1.53E-12 | 2.72E-10 |
| CDC42EP3 | -1.86408 | 8.312141 | 1.58E-12 | 2.75E-10 |
| CD83 | 2.668048 | 11.11535 | 1.58E-12 | 2.75E-10 |
| TGFB3 | 3.070183 | 7.798029 | 1.75E-12 | 3.01E-10 |
| ADRB2 | -1.62726 | 8.947002 | 1.83E-12 | 3.11E-10 |
| TRIB1 | 2.732567 | 9.595461 | 1.94E-12 | 3.24E-10 |
| PIM3 | 2.068378 | 8.942754 | 2.02E-12 | 3.35E-10 |
| PTAFR | -1.31028 | 12.00709 | 2.07E-12 | 3.39E-10 |
| ATMIN | 1.276006 | 10.81287 | 2.11E-12 | 3.41E-10 |
| BTG1 | 1.772691 | 11.34617 | 2.24E-12 | 3.57E-10 |
| PHLDA1 | 2.579935 | 7.25128 | 2.52E-12 | 3.98E-10 |
| NFKBIE | 1.635793 | 10.56161 | 2.67E-12 | 4.18E-10 |
| LRP4 | 1.416081 | 9.248327 | 2.73E-12 | 4.19E-10 |
| ZFP821 | 1.657946 | 8.955105 | 2.76E-12 | 4.19E-10 |
| SLC25A28 | 1.565418 | 9.765981 | 2.77E-12 | 4.19E-10 |
| VPS37B | 1.97633 | 10.15793 | 3.16E-12 | 4.72E-10 |
| ISY1 | 2.184988 | 10.57415 | 3.27E-12 | 4.83E-10 |
| TREM1 | 2.835391 | 7.904727 | 3.90E-12 | 5.71E-10 |
| SMIM3 | 1.88331 | 10.28184 | 4.01E-12 | 5.81E-10 |
| SPRED2 | 1.749166 | 9.068237 | 4.08E-12 | 5.85E-10 |
| SNX18 | 1.47967 | 10.78712 | 4.17E-12 | 5.92E-10 |
| ARL4C | 1.398414 | 12.52847 | 4.57E-12 | 6.41E-10 |
| RNF125 | 1.698496 | 5.772521 | 4.62E-12 | 6.43E-10 |
