# Supplementary material for: Norepinephrine promotes triglyceride storage in macrophages via beta2‐adrenergic receptor activation
Source: FASEB J. 2021 Jan 23;35(2):e21266. doi: 10.1096/fj.202001101R (PMC7898725; doi:10.1096/fj.202001101R)
Supplement: Supplementary file 7 — Table S2 [file FSB2-35-e21266-s001.docx]

**Supplementary table 2.** Top 100 differentially expressed genes between BMDMs treated with 1 μM fenoterol for 4 hours and untreated controls, ranked based on adjusted p value of change.

| **Gene name** | **Log(Fold change)** | **Average expression** | **p value** | **Adjusted p value** |
| --- | --- | --- | --- | --- |
| THBS1 | 6.973356 | 15.37731 | 1.82E-18 | 1.40E-14 |
| TGM2 | 4.538 | 12.97633 | 2.02E-18 | 1.40E-14 |
| CYTIP | 4.510348 | 11.87469 | 4.56E-18 | 2.11E-14 |
| FOSL2 | 3.849189 | 12.29506 | 1.10E-16 | 3.81E-13 |
| DUSP5 | 4.907857 | 9.893106 | 2.34E-16 | 6.50E-13 |
| GPR155 | -3.54076 | 8.273243 | 3.95E-16 | 9.15E-13 |
| GCSH | 3.152363 | 10.77913 | 5.01E-16 | 9.95E-13 |
| SLC16A3 | 3.966439 | 11.96858 | 9.34E-16 | 1.62E-12 |
| FAM214A | -3.53309 | 8.557312 | 1.30E-15 | 2.01E-12 |
| TREM1 | 4.57371 | 7.904727 | 2.49E-15 | 3.46E-12 |
| CYTH1 | 2.265148 | 12.22054 | 3.98E-15 | 4.87E-12 |
| LMO2 | -1.89572 | 10.99653 | 4.20E-15 | 4.87E-12 |
| EHD4 | -1.90332 | 12.87851 | 6.63E-15 | 7.09E-12 |
| ISY1 | 3.248167 | 10.57415 | 7.38E-15 | 7.33E-12 |
| TNS3 | -2.31687 | 12.30224 | 9.64E-15 | 8.63E-12 |
| CGNL1 | 3.273171 | 10.44729 | 9.93E-15 | 8.63E-12 |
| STAT4 | 2.897621 | 8.175092 | 1.19E-14 | 9.73E-12 |
| PLAUR | 2.375382 | 11.35404 | 2.00E-14 | 1.53E-11 |
| CD14 | 2.525057 | 15.20939 | 2.13E-14 | 1.53E-11 |
| PDE4D | 2.820311 | 8.225374 | 2.36E-14 | 1.53E-11 |
| ABCB1A | 3.252288 | 7.454528 | 2.42E-14 | 1.53E-11 |
| TNFRSF23 | 2.336087 | 9.067724 | 2.46E-14 | 1.53E-11 |
| ARHGAP17 | -1.74026 | 11.47075 | 2.53E-14 | 1.53E-11 |
| RAB44 | 4.778699 | 8.164872 | 2.68E-14 | 1.55E-11 |
| FGD4 | -1.97988 | 10.54642 | 2.92E-14 | 1.62E-11 |
| SLC4A11 | 3.206611 | 7.2149 | 3.09E-14 | 1.65E-11 |
| CD48 | -1.69044 | 11.54418 | 3.20E-14 | 1.65E-11 |
| CFAP43 | 1.985832 | 10.23769 | 3.86E-14 | 1.90E-11 |
| TTC39B | 2.612135 | 10.0439 | 3.97E-14 | 1.90E-11 |
| B3GALNT1 | -1.98965 | 9.077299 | 4.76E-14 | 2.21E-11 |
| VDR | 3.31336 | 7.116395 | 5.14E-14 | 2.25E-11 |
| **DGAT1** | 3.325186 | 11.08892 | 5.34E-14 | 2.25E-11 |
| MXI1 | 1.947385 | 11.60536 | 5.47E-14 | 2.25E-11 |
| IL1R1 | 3.813751 | 7.432322 | 5.65E-14 | 2.25E-11 |
| KIF3A | -1.93702 | 10.482 | 5.67E-14 | 2.25E-11 |
| NR4A2 | 3.60059 | 9.04992 | 5.96E-14 | 2.30E-11 |
| NID2 | 2.472395 | 8.155357 | 6.56E-14 | 2.46E-11 |
| KLHL6 | -2.01851 | 9.320247 | 6.85E-14 | 2.51E-11 |
| SIPA1L2 | 1.776783 | 10.57517 | 8.37E-14 | 2.98E-11 |
| UCK2 | 3.14022 | 10.00241 | 9.50E-14 | 3.30E-11 |
| SLC15A4 | -1.73207 | 9.89221 | 1.05E-13 | 3.55E-11 |
| ID3 | 2.941557 | 8.941129 | 1.11E-13 | 3.69E-11 |
| PTPRA | -1.42531 | 12.26595 | 1.27E-13 | 4.11E-11 |
| CREM | 3.947411 | 11.14653 | 1.43E-13 | 4.53E-11 |
| CNNM2 | 2.383746 | 10.32725 | 1.65E-13 | 5.09E-11 |
| SESN1 | -2.39326 | 11.13755 | 1.75E-13 | 5.27E-11 |
| NDRG1 | -2.36641 | 10.70817 | 1.78E-13 | 5.27E-11 |
| ETL4 | -2.38066 | 7.833028 | 1.96E-13 | 5.62E-11 |
| CASS4 | 3.044399 | 10.45168 | 1.98E-13 | 5.62E-11 |
| PDE8A | 1.605552 | 10.89749 | 2.14E-13 | 5.96E-11 |
| GOT1 | 2.013166 | 12.05794 | 2.33E-13 | 6.36E-11 |
| THAP6 | 2.5959 | 9.91523 | 2.42E-13 | 6.46E-11 |
| CMAH | -2.09808 | 6.979799 | 2.58E-13 | 6.66E-11 |
| ADAM8 | 2.346058 | 15.09188 | 2.59E-13 | 6.66E-11 |
| MAPRE2 | -1.52003 | 11.01269 | 2.77E-13 | 7.00E-11 |
| AU020206 | -1.76679 | 8.715366 | 2.82E-13 | 7.01E-11 |
| FOS | 2.852825 | 12.09094 | 3.01E-13 | 7.33E-11 |
| WWP1 | -1.84621 | 12.8118 | 3.15E-13 | 7.54E-11 |
| TLR5 | 3.937484 | 8.459243 | 3.47E-13 | 8.13E-11 |
| TJP2 | 2.190745 | 9.985094 | 3.51E-13 | 8.13E-11 |
| AI839979 | 1.758186 | 7.98878 | 3.68E-13 | 8.39E-11 |
| SIPA1L1 | 2.460248 | 11.24799 | 3.98E-13 | 8.91E-11 |
| RAB11FIP1 | 1.801667 | 9.850896 | 4.09E-13 | 9.02E-11 |
| B4GALT5 | 1.221635 | 12.72327 | 4.52E-13 | 9.70E-11 |
| PITPNC1 | 2.215773 | 11.63444 | 4.53E-13 | 9.70E-11 |
| ANTXR2 | 2.168011 | 12.90479 | 4.69E-13 | 9.78E-11 |
| PLEKHM1 | -1.76032 | 11.72529 | 4.72E-13 | 9.78E-11 |
| **HILPDA** | 2.544565 | 9.186574 | 4.79E-13 | 9.79E-11 |
| SLC45A4 | -1.70651 | 10.16226 | 5.20E-13 | 1.05E-10 |
| MAN1C1 | -1.45165 | 11.79068 | 5.34E-13 | 1.06E-10 |
| GPD2 | 1.838848 | 10.12749 | 6.09E-13 | 1.19E-10 |
| GYG | -1.55185 | 11.04045 | 6.61E-13 | 1.28E-10 |
| RNASEL | -1.85678 | 11.53483 | 6.92E-13 | 1.32E-10 |
| NT5E | 3.97704 | 6.824931 | 7.81E-13 | 1.47E-10 |
| CEBPA | -1.4089 | 10.35153 | 8.43E-13 | 1.56E-10 |
| MITF | -1.75373 | 10.29268 | 9.50E-13 | 1.70E-10 |
| PDE3B | 2.875219 | 10.85847 | 9.51E-13 | 1.70E-10 |
| ADORA2B | 3.253166 | 8.306417 | 9.55E-13 | 1.70E-10 |
| MPP1 | -1.44881 | 12.68997 | 1.04E-12 | 1.83E-10 |
| GPR35 | 3.936776 | 8.28316 | 1.09E-12 | 1.89E-10 |
| PTPRF | 2.59255 | 6.439259 | 1.27E-12 | 2.18E-10 |
| ATP6V0A1 | -1.54454 | 13.06542 | 1.30E-12 | 2.20E-10 |
| RRP1B | 1.696225 | 9.088756 | 1.46E-12 | 2.40E-10 |
| POGK | -1.41445 | 8.913897 | 1.46E-12 | 2.40E-10 |
| GAB3 | -1.39489 | 8.347776 | 1.47E-12 | 2.40E-10 |
| DMKN | 2.152516 | 6.364744 | 1.63E-12 | 2.64E-10 |
| LPAR1 | 1.761596 | 6.594138 | 1.67E-12 | 2.66E-10 |
| FAM20C | -1.53556 | 11.28091 | 1.73E-12 | 2.73E-10 |
| ELK3 | 1.26823 | 11.12405 | 1.89E-12 | 2.96E-10 |
| KCNJ2 | -2.60978 | 7.888283 | 1.96E-12 | 3.02E-10 |
| IMPDH1 | 1.423718 | 10.70942 | 2.15E-12 | 3.29E-10 |
| SRGAP2 | -1.26744 | 11.78289 | 2.24E-12 | 3.38E-10 |
| FAM49A | -2.29419 | 9.762266 | 2.29E-12 | 3.39E-10 |
| SOCS6 | -1.8538 | 9.202949 | 2.29E-12 | 3.39E-10 |
| PRKCH | -1.61811 | 10.20728 | 2.57E-12 | 3.70E-10 |
| DMWD | 2.302322 | 8.423696 | 2.58E-12 | 3.70E-10 |
| YWHAH | 1.572954 | 11.58673 | 2.58E-12 | 3.70E-10 |
| ETS2 | 1.878852 | 11.08148 | 2.87E-12 | 4.07E-10 |
| ETV3 | -1.32336 | 11.13014 | 3.07E-12 | 4.31E-10 |
| CITED2 | -1.39807 | 9.029236 | 3.11E-12 | 4.33E-10 |
